# The Somatic Mutation Landscape of UDP-Glycosyltransferase (*UGT*) Genes in Human Cancers

**DOI:** 10.3390/cancers14225708

**Published:** 2022-11-21

**Authors:** Dong Gui Hu, Shashikanth Marri, Julie-Ann Hulin, Ross A. McKinnon, Peter I. Mackenzie, Robyn Meech

**Affiliations:** College of Medicine and Public Health, Flinders Health and Medical Research Institute, Flinders University, Bedford Park 5042, Australia

**Keywords:** UDP-glycosyltransferase, UDP-glucuronosyltransferase, cancer, somatic mutation, drug metabolism, biomarkers

## Abstract

**Simple Summary:**

The human UDP-glycosyltransferase (UGT) superfamily is involved in the metabolism of numerous anticancer drugs and endobiotic signaling molecules with pro/anti-cancer activities. Previous studies have shown abundant expression of *UGT* genes in many human cancers, indicative of the active intratumoral metabolism of drugs and endobiotics through the UGT conjugation pathway. Mutations of *UGT* genes in tumors that can affect this pathway have not yet been reported. In the present study, our analysis of somatic mutations in 10,069 tumors from 33 different cancer types identified 3427 somatic mutations in *UGT* genes, over half of which have been predicted to code for variant UGT proteins with no or reduced activity. As a result, somatic mutations of *UGT* genes may reduce the capacity of cancer cells to metabolize anticancer drugs and pro/anti-cancer endobiotics, and hence, they are likely to alter therapeutic efficacy and cancer growth, highlighting their potential utility as biomarkers predicting therapeutic efficacy and clinical outcomes.

**Abstract:**

The human UDP-glycosyltransferase (UGTs) superfamily has a critical role in the metabolism of anticancer drugs and numerous pro/anti-cancer molecules (e.g., steroids, lipids, fatty acids, bile acids and carcinogens). Recent studies have shown wide and abundant expression of *UGT* genes in human cancers. However, the extent to which *UGT* genes acquire somatic mutations within tumors remains to be systematically investigated. In the present study, our comprehensive analysis of the somatic mutation profiles of 10,069 tumors from 33 different TCGA cancer types identified 3427 somatic mutations in *UGT* genes. Overall, nearly 18% (1802/10,069) of the assessed tumors had mutations in *UGT* genes with huge variations in mutation frequency across different cancer types, ranging from over 25% in five cancers (COAD, LUAD, LUSC, SKCM and UCSC) to less than 5% in eight cancers (LAML, MESO, PCPG, PAAD, PRAD, TGCT, THYM and UVM). All 22 *UGT* genes showed somatic mutations in tumors, with UGT2B4, UGT3A1 and UGT3A2 showing the largest number of mutations (289, 307 and 255 mutations, respectively). Nearly 65% (2260/3427) of the mutations were missense, frame-shift and nonsense mutations that have been predicted to code for variant UGT proteins. Furthermore, about 10% (362/3427) of the mutations occurred in non-coding regions (5′ UTR, 3′ UTR and splice sites) that may be able to alter the efficiency of translation initiation, miRNA regulation or the splicing of UGT transcripts. In conclusion, our data show widespread somatic mutations of *UGT* genes in human cancers that may affect the capacity of cancer cells to metabolize anticancer drugs and endobiotics that control pro/anti-cancer signaling pathways. This highlights their potential utility as biomarkers for predicting therapeutic efficacy and clinical outcomes.

## 1. Introduction

The human UDP-glycosyltransferase (*UGT*) superfamily comprises four subfamilies (*UGT1*, *UGT2*, *UGT3* and *UGT8*) that code for 22 functional UGT enzymes [1,2]. UGTs conjugate small lipophilic compounds with UDP-sugars to generate water-soluble products, thus facilitating their excretion from the body [3]. The nine UGT1 (1A1, 1A3–1A10) and ten UGT2 (2A1, 2A2, 2A3, 2B4, 2B7, 2B10, 2B11, 2B15, 2B17 and 2B28) enzymes primarily use UDP-glucuronic acid to conjugate therapeutic drugs and numerous endogenous (e.g., steroid hormones, bile acids, bilirubin and fatty acids) and exogenous (e.g., carcinogens, dietary constituents and environmental toxins) compounds and are hence traditionally termed UDP-glucuronosyltransferases [3,4,5]. By contrast, the UGT3 and UGT8 enzymes use differing UDP-sugars as donors, including UDP-N-acetylglucosamine (UGT3A1), UDP-glucose/UDP-xylose (UGT3A2) and UDP-galactose (UGT8) [6,7,8]. Their substrates include endogenous molecules such as bile acids (UGT3A1 and UGT8), ceramide (UGT8) and some xenobiotics, such as polycyclic aromatic hydrocarbons (UGT3A1 and UGT3A2) [6,7,8,9].

UGTs metabolize a variety of anticancer drugs (e.g., irinotecan and epirubicin) and thus affect their efficacy and toxicity [10,11,12,13,14,15]. For example, low-activity *UGT1A1* alleles (e.g., UGT1A1*28 and UGT1A1*6) are associated with an increased risk for severe or life-threatening neutropenia and myelosuppression during and after irinotecan administration [10,16,17,18,19,20,21]. UGTs also play an important role in cancer development and progression through inactivating and clearing pro/anti-tumor signaling molecules, including carcinogens, bile acids, fatty acids and steroid hormones [5,22,23]. For example, the polymorphic UGT2B17-deleted allele (lacking UGT2B17 activity) is implicated in prostate carcinogenesis [24,25,26,27,28]. In prostate cancer, high UGT2B17 expression is associated with an increased Gleason score and risk of metastasis, CRPC progression and recurrence after prostatectomy [29,30,31,32,33,34].

*UGT* genes are highly expressed in drug-metabolizing tissues and organs (e.g., the liver, intestine and kidney), consistent with their major role in systemic drug metabolism [3,4]. *UGT* genes are also widely expressed in many non-drug-metabolizing tissues, supporting their role in drug metabolism by peripheral tissues [4,31,35,36,37,38]. Many studies have further shown abundant expression of *UGT* genes in a variety of human cancers and their associations with tumor progression and recurrence as well as patient survival, highlighting the impact of the intratumoral metabolism of anticancer drugs and other pro/anti-cancer signaling molecules through the UGT conjugation pathway [22,29,30,39,40,41,42,43,44,45,46]. The deregulation of *UGT* gene expression and somatic mutations that alter UGT activity within cancer cells could affect this pathway. The deregulation of *UGT* genes resulting in increased or decreased expression in many cancers has been reported [31,40,41,42,43,44,47,48]; however, somatic mutations of *UGT* genes in cancers have not yet been systematically investigated.

Assessing genome-wide somatic mutation profiles or somatic mutations in genes of interest in human cancers has been the subject of numerous studies over the last few decades. Using whole exome sequencing of tumor-normal pairs, the Multi-Center Mutation Calling in Multiple Cancers (MC3) project recently analyzed over 10,000 tumors from 33 different TCGA (the Cancer Genome Atlas) cancer types and reported about 3.5 million somatic mutations [49]. The resulting mutation data (i.e., the MC3 MAF file) has formed the basis for many recent TCGA PanCan Atlas studies that have assessed somatic mutations for specific genes or groups of genes involved in specific signaling pathways [50,51,52]. Cancer cell lines are derived from tumor tissues and are frequently used as experimental models for the study of cancer biology and therapy [53]. Cancer cell lines contain mutations from the original tumors and inevitably acquire additional mutations through in vitro cultures. The Cancer Cell Line Encyclopedia (CCLE) project recently characterized the genome-wide mutation profiles for over 1500 human cancer cell lines (https://depmap.org) (accessed on 1 May 2022) [54]. In the present study, we comprehensively assessed the mutation profiles of *UGT* genes from both the MC3 and CCLE mutation datasets, and we report for the first time the mutation landscape of *UGT* genes in human cancers and cancer cell lines. We further discuss the potential impact and clinical implications of somatic mutations in *UGT* genes on the capacity of cancer cells to metabolize anticancer drugs and pro/anti-cancer signaling molecules through the UGT conjugation pathway.

## 2. Materials and Methods

### 2.1. Assessment of Somatic Mutations of UGT Genes in Human Cancers

#### 2.1.1. The Multi-Center Mutation Calling in Multiple Cancers (MC3) Project

To enable robust across-cancer-type analyses, the MC3 project assessed the whole exome sequencing data of 10,510 tumor-normal pairs from 33 TCGA cancer types using a uniform set of seven well-proven mutation-calling algorithms, including Indelocator, MuSE, MuTect, Pindel, RADIA, SomaticSniper and VarScan [49,55,56,57,58,59,60]. The resulting dataset is aggregated in Mutation Annotation Format (MAF) (https://docs.gdc.cancer.gov/Encyclopedia/pages/Mutation_Annotation_Format_TCGAv2) (accessed on 1 March 2022) and is publicly available from the NCI Genomic Data Commons (GDC), including protected Variant Call Format (VCF) file releases and a filtered, open-access TCGA MC3 MAF release that contains only the highest-confidence somatic mutations in exonic regions of protein-coding genes [49] (https://gdc.cancer.gov/about-data/publications/pancanatlas) (accessed on 1 March 2022). The MC3 MAF file lists 3,600,963 somatic mutations from 10,295 tumors, including 3,427,680 point mutations (single nucleotide variants, SNV) and 173,283 small deletions and insertions (indels) [49]. In the present study, we used the MC3 MAF file (mc3.v0.2.8.PUBLIC.maf.gz) to identify the somatic mutations of *UGT* genes in human cancers, as described in detail below. 

#### 2.1.2. Extracting Individual Somatic Mutations from the MC3 MAF File and Assigning Them to Each of the 33 TCGA Cancer Types

The MC3 MAF file lists all somatic mutations from 10,295 tumors alphabetically and numerically according to the TCGA barcodes of the tumor samples (mc3.v0.2.8.PUBLIC.maf.gz). Therefore, the entries are grouped by tumor sample and not by cancer type. We manually allocated the tumor samples from the MC3 MAF file into each of the 33 TCGA cancer types. Consistent with the primary aim of the TCGA project focusing on the study of primary tumors, the majority of samples were primary tumors; however, many cancer types also contained recurrent (BRCA, COAD, GBM, LGG, LIHC, LUAD, READ and SARC) and metastatic (BRCA, CESC, COAD, LGG, PAAD, PRAD and PCPG) tumor samples. To ensure a consistent analysis of only primary tumors within and between cancer types, all recurrent tumors were excluded from the analysis. Metastatic tumors were also excluded from the analysis for all cancer types except SKCM. The SKCM cohort contained about 75% metastatic tumors and 25% primary tumors [61,62]. In the present study, we assessed metastatic SKCM tumors (364 samples) as a sub-cohort (designated as Metastatic SKCM) as compared with primary SKCM tumors (103 samples) (designated as Primary SKCM) (Table 1, Table S1).

The TCGA PAAD (Pancreatic Adenocarcinoma) cohort from several repositories contained samples that were not primary PAAD [63,64]. The inclusion of these misclassified samples in the analysis significantly skewed the association of molecular biomarkers with clinical outcomes [63,64]. Similarly, the PAAD cohort from the MC3 MAF file had 23 misclassified samples, including (1) pseudonormal samples with <1% neoplastic cellularity, (2) tumors not derived from the pancreas, (3) neuroendocrine tumors, (4) acinar cell carcinoma, (5) intraductal papillary mucinous neoplasm, (6) metastatic tumors, (7) undifferentiated tumors and (8) systemically treated tumors (Table S1). In the present study, we excluded these samples from the analysis of the PAAD cohort.

Most tumors have a relatively low mutation burden; however, tumors with extremely high numbers of somatic mutations have been reported for many cancers, such as melanoma, lung, endometrial and bladder cancer [49,65,66,67,68]. This hypermutation can be caused by genetic defects (e.g., replication repair defects) [69,70], mutagen exposure (e.g., UV lights and tobacco smoking) [71,72] and anticancer therapy (e.g., immune checkpoint inhibitors) [73,74,75]. Hypermutated tumors are very rare in cancers with a low mutation burden and only account for a very small portion of tumors for cancers with a high mutation burden. Therefore, it was necessary to exclude hypermutated tumors from the analysis to avoid their potential influence on the results [49,52]. In the present study, we excluded 53 hypermutated tumors from the analysis, including tumors from cancers with a high (COAD, SKCM, STAD and UCEC) or low (GBM, LGG, CESC, PAAD, PRAD and UCS) mutation load (Table S1). Therefore, 878,185 mutations (16,569 mutations on average per tumor) from these hypermutated tumors were excluded from the analysis (Table S1). 

Collectively, after having excluded recurrent, metastatic (except SKCM) and hypermutated tumors, we obtained 9705 primary tumors and 364 metastatic SKCM tumors from the MC3 MAF file that together had 2,686,092 somatic mutations (Table 1). Table S1 lists the tumors that were analyzed in this study for each of the 33 TCGA cancer types.

#### 2.1.3. Verifying and/or Correcting the Assignments of Individual Somatic Mutations from the MC3 MAF to Each of the 22 *UGT* Genes 

The MC3 MAF file is a tab-delimited text file that contains comprehensive information for each mutation, including the mutated gene (Hugo_Symbol) and the positions of the mutation at the genomic (GRCh37/hg19), cDNA (Ensembl Reference Transcripts) and protein levels (Table S3). The mutations at eleven *UGTs* (*2A3*, *2B4*, *2B7*, *2B10*, *2B11*, *2B15*, *2B17*, *2B28*, *3A1*, *3A2* and *UGT8*) were clearly identified in the MC3 MAF file by the HUGO gene names (Hugo_Symbol). However, conflicting allocation and mis-annotation were seen in the MC3 MAF file for mutations for the remaining eleven *UGT* genes (nine *UGT1As*, *UGT2A1* and *UGT2A2*) primarily due to the exon-sharing genomic structure among *UGT1As* or *UGT2As* [1]. The nine *UGT1A* (*1A1*, *1A3–1A10*) genes have unique exon 1s and a shared set of exons 2–5 [1]. As expected, the MC3 MAF file lists mutations at the unique exon 1s for the respective *UGT1As*, but mutations within *UGT1A1* exon 1 are mis-annotated as intronic mutations for *UGT1A8*. We recalculated the positions at both the cDNA and protein levels for these mis-allocated mutations based on the UGT1A1 reference sequences (RefSeq: NM_000463, NP_000454) (designated as mutations for *1A1*) (Table 2). All mutations within the shared exons 2–5 affect all nine *UGT1As*, but these mutations are listed in the MC3 MAF file specifically as mutations for *UGT1A10* (exons 2–4) or *UGT1A4* (exon 5). In the present study, we correctly assigned these mutations to all nine *UGT1A*s (designated as mutations for *1A* E2–5) (Table 2). The *DNAJB3* gene [DnaJ (Hsp40) homolog, subfamily B, member 3)] is located between the first exons of *UGT1A1* and *UGT1A3*. There are 122 mutations at *DNAJB3* that are mis-annotated in the MC3 MAF file as intronic mutations for *UGT1A10* (Table S4). We excluded these mutations from the analysis of this study.

Similarly, *UGT2A1* and *UGT2A2* have different exon 1s and a common set of exons 2–6, and therefore, mutations within exons 2–6 affect both genes [1]. As expected, the MC3 MAF file lists the mutations at the unique exon 1s as mutations for the respective *UGT2A1* and *UGT2A2*; however, the mutations within the shared exons 2–6 are annotated in the MC3 MAF file using a variant UGT2A1 transcript (ENST00000514019, NM_001389565.1) that has the *UGT2A2* exon 1 inserted between *UGT2A1* exons 1 and 2. This insertion generates an extended 737-aa variant UGT2A1 protein (NP_001376494.1) as compared with the 527-aa wildtype UGT2A1 protein (NP_006789.3). In the present study, we recalculated the positions at both the cDNA and protein levels for these mis-annotated mutations based on the UGT2A1 NCBI reference sequences (RefSeq NM_006798, NP_006789). These mutations affect both *UGT2A1* and *UGT2A*2, and hence they are listed as “2A1/2A2 E2–6” in Table 2.

After identifying the mutations for each *UGT* gene, we manually assessed whether the annotated positions at the genomic, cDNA and protein levels are accurate and consistent for each of the mutations based on the NCBI GRCh37/hg19 reference sequences. Through this process, we were able to verify the accuracy of annotations for all mutations, but five mutations showed conflicting cDNA and genomic positions, including one mutation from each of three genes (*1A4*, *1A10*, *2B28*) and two mutations from *UGT1A5* (Table S4). We excluded these five mutations from the analysis of this study.

Collectively, we identified 1802 tumors from 33 TCGA cancer types in the MC3 MAF file that each have at least one mutation in a *UGT* gene (Table S2). These tumors together have 3427 somatic mutations in *UGT* genes that were included in the analysis of this study (Table 1 and Table 2). Table S3 lists the mutations in *UGT* genes for each of the 33 TCGA cancer types. Table S4 lists the mutations for each of the 22 *UGT* genes. 

To assess whether mutations in *UGT* genes are correlated with mutations in the tumor suppressor gene *TP53*, we determined the numbers of mutations in *TP53* for each of the 33 TCGA cancer types (Table S4).

The MC3 MAF file (variant-Classification column) classifies mutations into at least 13 different types of mutations according to the positions and nature of the mutations, including (1) mutations in 5′ or 3′ untranslated regions (5′UTR and 3′UTR), (2) mutations in coding regions (translation_start_site, missense, nonsense, silent, nonstop_mutation, frame_shift_del, frame_shift_in, in_frame_del and in_frame_in) and (3) mutations within introns and splice sites. The MC3 MAF file includes an assessment of all missense mutations by the SIFT (Sorting Intolerant From Tolerant) algorithm, which uses amino acid sequence homology to predict whether a missense substitution affects protein function, and it classifies missense substitutions as tolerated or deleterious [76,77]. Table 2 lists the number of each type of mutations for each of the 22 *UGT* genes, including the number of deleterious missense mutations. 

Using the Clustal Omega multiple sequence alignment program from the EMBL-EBI sequence analysis toolkit [78], we identified amino acids that are conserved across the UGT enzyme family and subfamilies, and we annotated mutations that affect conserved amino acids in multiple UGT proteins. To further highlight the mutations (i.e., missense, nonsense, nonstop and small indels) within coding sequences that may affect enzyme function, we mapped them at the cDNA and protein levels to clearly show their distribution throughout the coding regions and potential mutation hotspot regions.

### 2.2. Assessment of Somatic Mutations of UGT Genes in Human Cancer Cell Lines

The Cancer Cell Line Encyclopedia (CCLE) project comprehensively characterizes the molecular profiles of over 1000 human cancer cell lines (Broad, 2019), including the mutation profiles (CCLE_mutations.csv) for 18,784 human genes in 1771 human cancer cell lines using whole exome sequencing [54,79]. The resulting dataset is available from the DepMap Public 22Q2 via the CCLE DepMap portal (https://depmap.org/portal) (accessed on 1 August 2002). This dataset is further elaborated with additional annotations for every mutation in the cBio cancer genomics portal (cBioPortal) (https://www.cbioportal.org) (accessed on 1 August 2022) [80]. We obtained the mutations of all *UGT* genes except for *UGT2A2* exon 1 from the cBioPortal (Table S5). In the cBioportal, mutations in the exons 2–6 shared by *UGT2A1* and *UGT2A2* are annotated using the UGT2A2 reference sequence (ENST00000457664). Mutations in *UGT2A2* exon 1 were found in the DepMap dataset, but they are annotated based on the same variant UGT2A1 transcript (ENST00000514019) as described above for the TCGA MC3 MAF file (Table S5). We recalculated the positions at the cDNA and protein levels for these mutations based on the UGT2A2 NCBI reference sequences (RefSeq NM_006798 and NP_006789). Silent mutations are listed in the DepMap portal dataset but are not present in the cBioPortal dataset. There are no mutations at untranslated regions (5′ UTR and 3′ UTR) in both the DepMap- and cBio-Portal datasets. Collectively, our analysis of 1568 CCLE cell lines identified 895 mutations in *UGT* genes (Table 3 and Table S5).

As classified by the cBioPortal (https://www.cbioportal.org) (accessed on 1 August 2022), the assessed 1568 CCLE cell lines are derived from a variety of human cancers (Table S5), including cancers of unknown primary tumors (24.6%), mixed cancer types (10.3%), non-small cell lung cancer (8.3%), esophagogastric cancer (6.1%), glioma (3.9%), colorectal cancer (3.9%), melanoma (3.9%), mature B-cell neoplasms (3.7%), breast cancer (3.6%), ovarian cancer (3.4%), pancreatic cancer (2.7%), leukemia (2.6%), hepatobiliary cancer (2.3%), B-lymphoblastic leukemia/lymphoma (1.8%), bladder cancer (1.8%), bone cancer (1.8%), endometrial cancer (1.8%), kidney cancer (1.3%), soft tissue sarcoma (1.2%), neuroblastoma (1.1%), blood cancer (1.0%), thyroid cancer (0.8%), Hodgkin lymphoma (0.7%), renal cell carcinoma (0.7%), mesothelioma (0.6%), prostate cancer (0.5%) and embryonal tumors (0.3%).

### 2.3. Statistical Analysis

The potential correlation between the number of mutations in all genes and the number of mutations in *UGT* genes per tumor across 33 different TCGA cancer types was assessed by Spearman ranking correlation analysis using GraphPad Prism (version 9.1.1) (GraphPad Software, San Diego, CA, USA). A *p* value of <0.05 was considered statistically significant.

## 3. Results

### 3.1. Somatic Mutations of Protein-Coding Genes in Human Cancers

Using the MC3 MAF file, our analysis of the mutation profiles of 10,069 tumors identified 2,686,092 somatic mutations within the exonic sequences of human protein-coding genes (Table 1). Table 1 lists the number of tumors assessed as well as the total number of somatic mutations identified for each of the 33 different cancer types. The number of mutations varied greatly across cancer types. Metastatic SKCM and PCPG had the highest (1204) and lowest (15) number of mutations per tumor, respectively (Table 1). These data are consistent with previous studies that have identified cancer types with a high mutation burden (BLCA, COAD, LUAD, LUSC, SKCM, STAD and UCEC) and those with a relatively low mutation burden (KICH, LAML, LGG, MESO, PCPG, PAAD, PRAD, TGCT, THCA, THYM and UVM) [49,65,66,67,68]. A genome-wide analysis of the MC3 somatic mutations was recently reported [49]. In the present study, we focused on the analysis of somatic mutations in *UGT* genes, as described in detail below.

### 3.2. Somatic Mutations in UGT Genes in Human Cancers

#### 3.2.1. Summary

This section briefly summarizes our overall findings on the somatic mutation landscape of the *UGT* gene superfamily. Detailed descriptions of the different types of mutations found in individual *UGT* genes are described in subsequent sections. 

**Figure 1:**
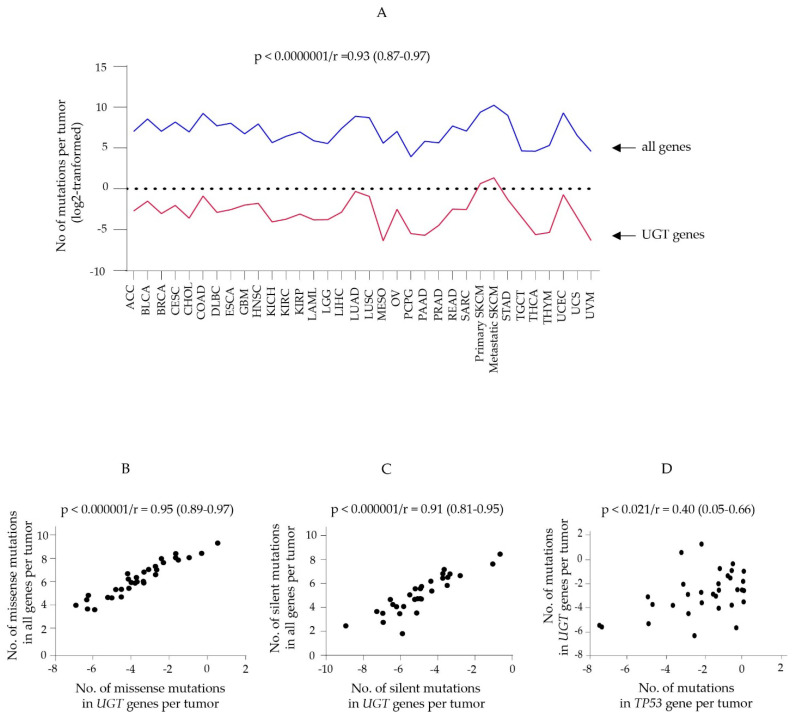
Assessment of the potential correlation between the numbers of mutations per tumor in *UGT* genes and in all protein-coding genes or *TP53* gene across 33 TCGA cancer types. The numbers of mutations per tumor in all protein-coding genes, *UGT* genes or *TP53* gene in 33 TCGA cancer types were log2− transformed and subjected to Spearman ranking correlation analysis using GraphPad Prism (9.1.1). (**A**–**C**) A diagram shows the correlation analysis between the numbers of all mutations (**A**), missense (**B**) or silent mutations (**C**) in all genes and in *UGT* genes. (**D**) A graph shows the correlation analysis between the numbers of all mutations in *UGT* genes and *TP53* gene. The *p* value, correlation coefficient (r) and 95% confidence interval of the correlation analyses are also shown. A *p* value of < 0.05 was considered statistically significant.

Of the assessed 10,069 tumors, 1802 tumors (17.8%) had at least one *UGT* gene mutation (Table 1). Together, these tumors had 3427 somatic mutations in *UGT* genes. Table 1 lists the number of tumors and the total number of mutations in *UGT* genes for each of the 33 cancer types. Overall, the total number of mutations in *UGT* genes per tumor varied widely across different cancer types and was positively correlated with the total number of mutations in all genes per tumor across cancer types (Spearman ranking correlation analysis: r = 0.939; *p* < 0.0000001) (Figure 1A). As described below, missense and silent mutations were the two most common types of mutations in *UGT* genes in TCGA tumors. We showed a positive correlation between the numbers of missense or silent mutations per tumor in *UGT* genes and in all genes across cancer types (Figure 1B,C). Collectively, these results indicate that the mutation rates of *UGT* genes in different types of cancers were defined by the differing mutation burdens of cancer types, as described in detail below. 

*TP53* is a tumor suppressor gene that is frequently mutated in TCGA tumors [81]. We showed a positive association between the total numbers of mutations per tumor in *TP53* gene and in *UGT* genes (Figure 1D). However, it remains to be investigated whether *TP53* influences *UGT* mutations or overall mutation burdens.

The majority of the 3427 mutations in *UGT* genes were found in several cancers with a high mutation burden, including 1086 mutations (31%) in SKCM tumors and 1500 mutations (43%) in six other cancers (BLCA, COAD, LUAD, LUSC, STAD and UCEC) (Table 1). By contrast, thirteen cancers with a low mutation burden had no (TGCT) or less than 15 (ACC, CHOL, DLBC, KICH, LAML, MESO, PCPG, PAAD, THCA, THYM, UCS and UVM) mutations in *UGT* genes (Table 1).

The percentage of tumor samples that had mutations in *UGT* genes also varied widely across cancer types. Over 20% of the tumors had mutations in *UGT* genes in nine cancers with a high mutation burden (BLCA, COAD, GBM, HNSC, LUAD, LUSC, SKCM, STAD and UCEC) (Table 1). Of these cancers, melanoma and lung cancer (SKCM-metastatic, SKCM-primary, LUAD and LUSC) had the highest frequencies (67.5%, 54.3%, 45.8% and 34.2%, respectively). By contrast, less than 1% of tumors had mutations in *UGT* genes in eight cancers with a low mutation burden (LAML, MESO, PCPG, PAAD, PRAD, THCA, THYM and UVM) (Table 1). 

Consistent with the positive correlation between the numbers of mutations per tumor in *UGT* genes and in all genes across cancer types (Figure 1A), cancer types with a high mutation burden had a higher frequency of tumors with multiple mutations in *UGT* genes. For example, the percentages of tumors with two or more mutations in *UGT* genes were 46% (166/364), 29% (30/103), 19% (99/512) and 10% (51/487) for SKCM-metastatic, SKCM-primary, LUAD and UCEC, respectively (Table S3). For example, among the 122 UCEC tumors with mutations in *UGT* genes, fifty-one had 2 (21 tumors), 3 (12 tumors), 4 (3 tumors), 5 (3 tumors), 6 (2 tumors), 7 (2 tumors), 8 (2 tumors), 10 (2 tumors), 13 (1 tumor), 14 (2 tumors) or 18 (1 tumor) mutations in *UGT* genes (Table S3). Multiple mutations within a tumor were generally in different *UGT* genes but were occasionally clustered in a single *UGT* gene. For example, the UCEC tumor (TCGA-D1-A17Q-01A-11D-A12J-09) had the largest number of mutations in *UGT* genes among UCEC tumors, including one mutation in each of eleven different *UGT* genes (*1A3*, *1A4*, *2A1*, *2A2*, *2B4*, *2B7*, *2B11*, *2B15*, *2B17*, *3A1* and *3A2*), three mutations in *UGT8*, and four mutations in *UGT2B28* (Table S3). 

The impact of mutations on protein function depends on the positions and nature of the mutations. The MC3 MAF file classified mutations into 13 different types, as described in the Materials and Methods Section [49]. All these types of mutations except “In_Frame_Ins” were found in *UGT* genes (Table 2). Table 2 lists the number of each type of mutation for each of the 22 *UGT* genes. Of the 3427 mutations in *UGT* genes, 3065 mutations were found in coding regions that were subclassified into eight different types of mutations (Table 2). Briefly, nearly a quarter of the mutations (754/3065) were silent mutations (synonymous mutations) that do not alter protein sequences. Nearly two-thirds of the mutations (1998/3065) were missense mutations resulting in amnio acid substitutions, approximately 55% (1099/1998) of which were defined by the SIFT algorithm as deleterious amino acid substitutions with a significant impact on UGT function [76]. Nonsense mutations resulting in premature stop codons accounted for about 6% (184/3065) of the mutations. Approximately 4% of the mutations (127/3065) were small deletions and insertions (Indels) that code for frame-shifted truncated proteins (frame_shift_del, frame_shift_ins) or variant proteins with small internal deletions (in_frame_del). Finally, one mutation within the start (*UGT1A6*) or stop (*UGT2B15*) codon was also observed. Mutations that introduce premature stop codons may lead to nonsense-mediated mRNA decay or may encode truncated proteins. Truncated UGTs generally have no transferase activity but might act as dominant negative regulators repressing UGT activity [82,83,84].

Although the MC3 project focused on the analysis of mutations in coding exonic regions of human protein-coding genes, we found that approximately 10% of the mutations in *UGT* genes (362/3427) occurred in untranslated regions (5′ UTR, 3′ UTR), introns and splice sites. These mutations do not change UGT protein sequences, but they may affect the splicing, stability and translation initiation of UGT transcripts, as described in detail below. 

#### 3.2.2. Mutations in the UGT1A Subfamily Genes

**Figure 2:**
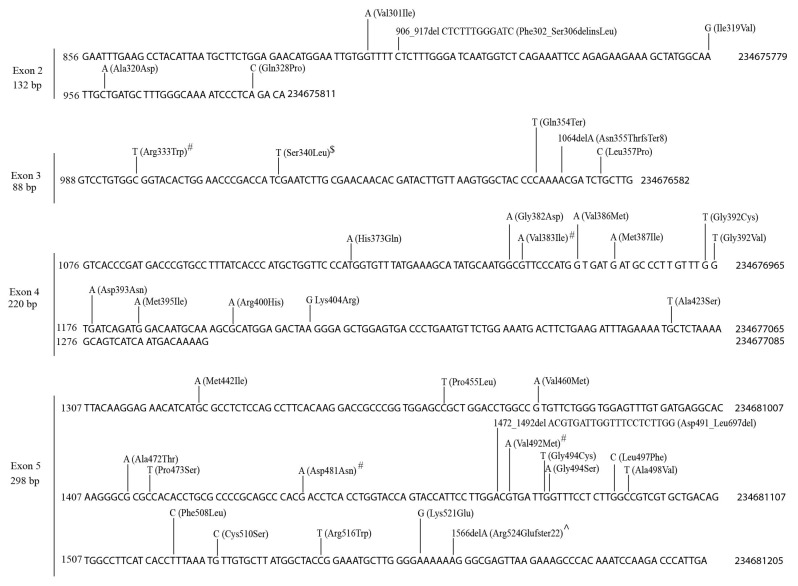
Mutations within the coding region of the shared *UGT1A* exons 2–5 in TCGA tumors. Data shown are the NCBI reference sequence (4 exons, 738 bp) for *UGT1A* exons 2–5 with genomic positions (GRCh37/hg19) indicated at the right, and the positions at cDNA (at left) and protein (above sequence) levels for exons 2–4 (defined by the UGT1A10 reference sequences: NM_019075.4 and NP_061948.1) and exon 5 (defined by the UGT1A4 reference sequences: NM_007120.3 and NP_009051.1). Mutations (missense, nonsense and small indels) and the resulting changes at the protein levels are indicated above the reference sequence. Recurrent mutations are indicated by # (twice), $ (three times) and ∧ (six times).

The *UGT1A* subfamily contains nine genes (*1A1*, *1A3–1A10*) which have unique first exons and a shared set of exons 2–5 [1]. We found 87 mutations within exons 2–5 and 68 to 96 mutations in the individual exon 1s in TCGA tumors (Table 2, Table S4). More than half of the mutations in exons 2–5 (Figure 2) and nine unique exon 1s (Figures S1–S9) were missense, nonsense or small indel mutations that result in amino acid substitutions or generate truncated proteins. Mutations in exons 2–5 affect all nine UGT1A enzymes; however, mutations in the first exon only affect the corresponding UGT1A enzyme. 

Most mutations in *UGT1A* genes generally occurred randomly throughout the coding sequences (Figure 2, Figures S1–S9). However, mutation hotspots were also observed. For example, there were 11 different mutations in a 23 bp region between nucleotides 663 and 685 of the *UGT1A9* exon 1 (Figure S8).

**Figure 3:**
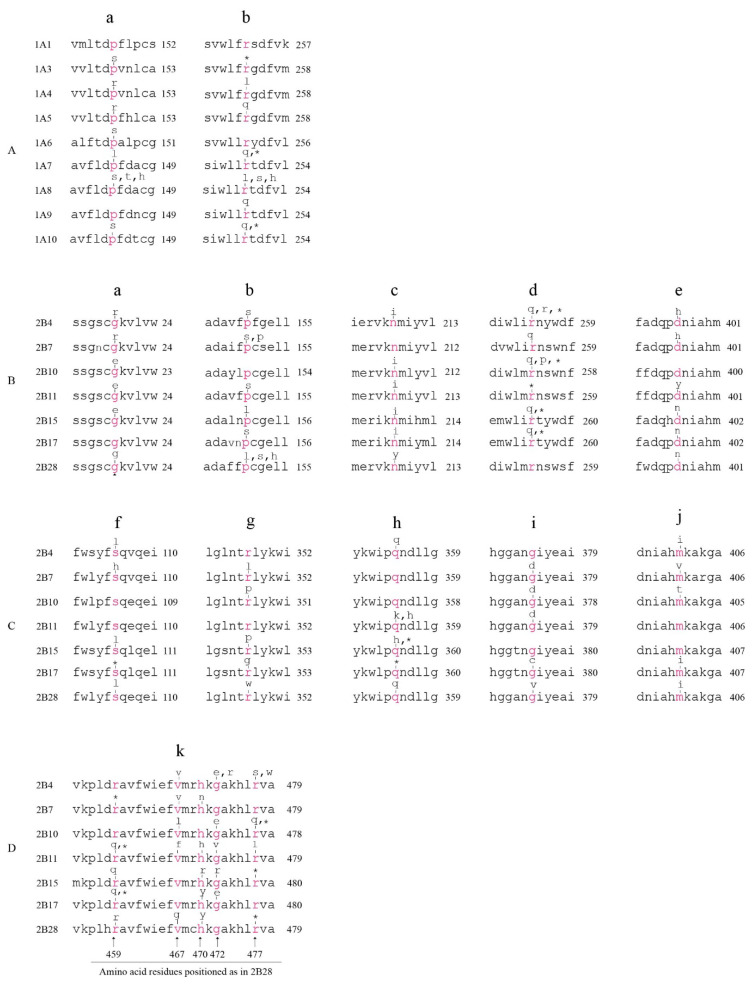
Mutations within the codons of conserved amino acids of the UGT1A and UGT2B family of enzymes. The amino acid sequences of the UGT1A enzymes (**A**) and UGT2B enzymes (**B**–**D**) are aligned by the Clustal Omega program. Data shown are the sequence alignments surrounding the conserved amino acids [in Red, positions given at the left for (**A**–**C**) or at the bottom for (**D**)] whose codons were mutated in TCGA tumors. (**A**) Mutations in the codons of UGT1A conserved amino acids (a,b). (**B**–**D**) Mutations in the codons of UGT2B conserved amino acids (a–k). As indicated above, for the conserved amino acids, these mutations lead to (1) amnio acid substitution (missense), (2) no change in amnio acid sequence (silent mutation) or (3) premature stop codons (nonstop mutation, specified by *).

The nine unique *UGT1A* exon 1s encode the N-terminal half (284–288 amino acids) of the UGT1A proteins. Using the Clustal Omega program, we identified 66 conserved amino acids within this region across all nine UGT1As. We identified two conserved amnio acids (^152^Pro and ^257^Arg as positioned in UGT1A1 protein sequence) whose codons were mutated in seven *UGT1A* genes, generating missense or nonsense mutations (Figure 3A). 

#### 3.2.3. Mutations in the UGT2A Subfamily of Genes

**Figure 4:**
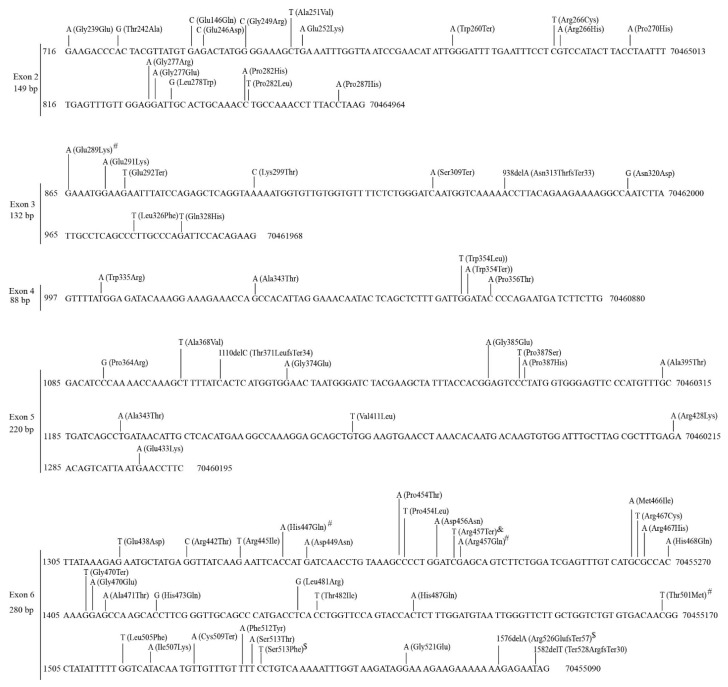
Mutations within the shared *UGT2A1/UGT2A2* exons 2–6 in TCGA tumors. Data shown are the NCBI reference sequence (5 exons, 869 bp) for *UGT2A1/2A2* exons 2–6 with genomic (GRCh37/hg19, right) and cDNA (NM_006798.5, left) positions. Mutations (missense, nonsense and small indels) and the resulting changes at the protein level (NP_006789.3) are indicated above the reference sequence. Recurrent mutations are indicated by # (twice), $ (three times) and & (five times).

The *UGT2A* subfamily contains three genes (*2A1*, *2A2*, *2A3*), two of which (*2A1*, *2A2*) have unique first exons and share exons 2–6 [3]. In TCGA tumors, we found 121 mutations within the shared exons 2–6 and 78 and 73 mutations in *UGT2A1* and *UGT2A2* exon 1s, respectively (Table 2, Table S4). There were 196 mutations in *UGT2A3* (Figure S12, Table 2*).* Overall, over 70% of these mutations were missense, nonsense or small indel mutations that lead to amino acid substitutions or produce truncated proteins. Mutations in *UGT2A1/2A2* exon 1s only influence the respective enzymes (Figures S10 and S11); however, mutations in the shared exons 2–6 affect both UGT2A1 and UGT2A2 (Figure 4).

Mutations generally occurred randomly throughout the coding sequences of the three *UGT2A* genes; however, within the shared *UGT2A1/2A2* exons 2–6, exons 2 and 6 appear to have more mutations than the three other exons, representing mutation hotspots (Figure 4).

#### 3.2.4. Mutations in the UGT2B Subfamily Genes

**Figure 5:**
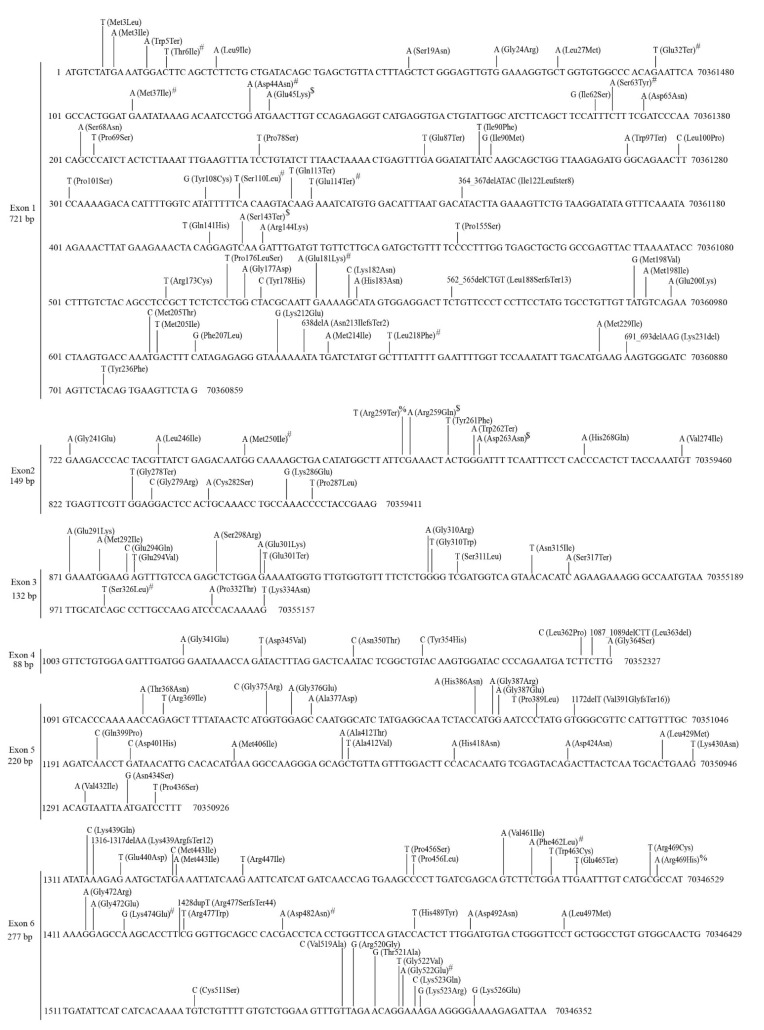
Mutations in the *UGT2B4* gene in TCGA cancers. Data shown are the NCBI UGT2B4 reference sequence (6 exons, 1587 bp) with genomic (GRCh37/hg19, right) and cDNA (NM_021139.3, left) positions. Mutations (missense, nonsense and small indels) and the resulting changes at the protein level (NP_066962.2) are indicated above the reference mRNA sequence. Recurrent mutations are indicated by # (twice), $ (three times) and % (four times).

The UGT2B gene subfamily comprises seven genes (2B4, 2B7, 2B10, 2B11, 2B15, 2B17 and 2B28) [1]. We found 289, 172, 193, 205, 210, 147 and 210 mutations in TCGA tumors in *UGT2B4*, *UGT2B7*, *UGT2B10*, *UGT2B11*, *UGT2B15*, *UGT2B17* and *UGT2B28*, respectively (Table 2, Table S4). Overall, approximately 60% of the mutations in *UGT2B4* (Figure 5) and the other six genes (Figures S13–S18) were missense mutations, nearly 60% of which were considered by the SIFT algorithm to be deleterious amino acid substitutions with a significant impact on protein function (Table 2). Furthermore, 15% of the mutations in *UGT2B* genes were nonsense or small indels that introduce premature stop codons or that could code for truncated proteins (Table 2). 

Mutations were generally present throughout the seven *UGT2B* genes (Figure 5, Figures S13–S18); however, there were many mutations clustered together to form hotspots. Three typical examples of such hotspots are: (1) seven mutations within a 13 bp region in *UGT2B4* (c.1556–1568) (Figure 5), (2) seven mutations within a 16 bp region in *UGT2B10* (c.1565–1580) (Figure S14) and (3) 12 mutations within a 31 bp region in *UGT2B28* (c.1153–1183) (Figure S18).

The UGT2B proteins each comprise 528–530 amino acids [1]. Using the Clustal Omega program, we identified 342 conserved amino acids across all seven UGT2B proteins. Mutations in multiple *UGT2B* genes that affect the same conserved amino acids were frequently observed. For example, the codons corresponding to five conserved amino acids (i.e., ^24^Gly, ^155^Pro, ^213^Asn, ^259^Arg and ^401^Asp, positioned in UGT2B4) were mutated in six *UGT2B* genes (Figure 3B). The codons of another ten conserved amino acids (i.e., ^110^Ser, ^352^Arg, ^359^Gln, ^379^Gly, ^406^Met, ^459^Arg, ^467^Val, ^470^His, ^472^Gly and ^477^Arg, positioned in UGT2B28) were mutated in five *UGT2B* genes (Figure 3C,D). Of note, five of these ten conserved amino acids (i.e., ^459^Arg, ^467^Val, ^470^His, ^472^Gly and ^477^Arg) cluster together within a highly conserved 19-amnio-acid region within the UGT2B proteins, implying a mutation hotspot (Figure 3D). 

The UGT1A and UGT2B proteins have similar sizes (528–534 amino acids) [1]. Using the Clustal Omega program, we identified 164 conserved amino acids across the UGT1A and UGT2B proteins, including ^259^Arg, ^359^Gln and ^472^Gly (as in UGT2B28) (Figure S19). Mutations in multiple *UGT1A* and *UGT2B* genes that affect the same conserved amino acids were frequently observed. For example, the codon for the conserved ^259^Arg was mutated in seven *UGT1A* genes (*1A3*, *1A4*, *1A5*, *1A7*, *1A8*, *1A9* and *1A10*) and six *UGT2B* genes (2B4, 2B7, 2B10, 2B11, 2B15 and 2B17) (Figure S19A), and the codons for the conserved ^359^Gln and ^472^Gly were mutated in all nine *UGT1A* and five *UGT2B* genes, implying mutation hotspots (Figure S19B,C). 

#### 3.2.5. Mutations in the UGT3 Subfamily Genes

The *UGT3* subfamily comprises *UGT3A1* and *UGT3A2* [1]. We found 307 and 255 somatic mutations in TCGA tumors in *UGT3A1* and *UGT3A2*, respectively (Table 2, Table S4). We found that 53% of the mutations (165/307) in *UGT3A1* and 62% of the mutations (159/255) in *UGT3A2* result in amino acid substitutions (missense), premature stop codons (nonsense) or frame-shift truncated proteins (small indels) (Figures S20 and S21, Table 2). Approximately 45% of the missense mutations in UGT3A1 and UGT3A2 were SIFT-defined as deleterious amino acid substitutions with a significant impact on protein function (Table 2). Most mutations occurred randomly throughout the two *UGT3A* genes; however, mutation hotspots with multiple mutations clustered together were also observed. Examples of such hotspots include (1) six mutations within an 11 bp region in *UGT3A1* (c.1396–1406) (Figure S20) and (2) six mutations within a 16 bp region in *UGT3A2* (c.17–32) (Figure S21).

#### 3.2.6. Mutations in the UGT8 Gene

We found 140 somatic mutations in *UGT8* in TCGA tumors, nearly 70% (97/140) of which were missense, nonsense and small indels that result in amino acid substitutions, premature stop codons and frame-shift truncated proteins, respectively (Table 2, Figure S22). Approximately half (47/89) of the missense mutations were defined by SIFT to be deleterious amino acid substitutions with a significant effect on protein function. Mutations were randomly distributed across the *UGT8* gene (Figure S22).

#### 3.2.7. Mutations in the 5′ UTRs of UGT Genes

**Figure 6:**
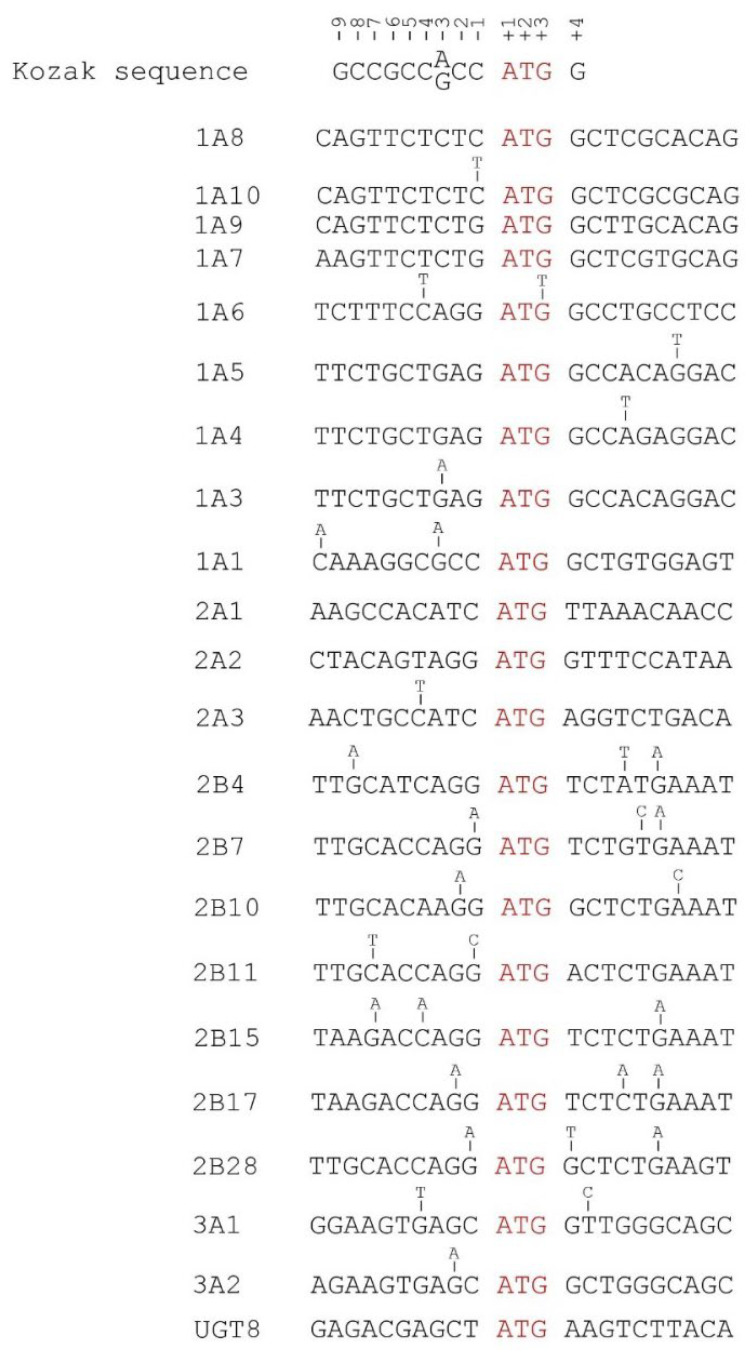
Somatic mutations within and adjacent to the Kozak sequence of *UGT* genes in TCGA tumors. Data shown are the Kozak consensus sequence and the start codon ATG (positioned as +1, +2 and +3) and 10 nucleotides up (positioned as −1 to −10)- and down (positioned as +4 to +13)-stream of 22 *UGT* genes. Mutations within the Kozak sequences (as defined from position −9 to position +4) and adjacent sequences of *UGT* genes are indicated above the sequences. The start codons of the 22 *UGT* genes are highlighted in RED.

The Kozak sequence [GCCGCC(A/G)CCAUGG] [(positioned as +1 for A in start codon AUG (underlined)] surrounding the start AUG codon is critical for translation initiation (Figure 6) [85,86,87]. An A or G in the −3 position and a G in the +4 position represent the optimal Kozak motif (Figure 6). Variations at all other positions have no or a weak impact on translation initiation [85]. Nine (i.e., *1A1*, *1A3*, *1A4*, *1A5*, *1A6*, *2B10*, *2B28*, *3A1* and *3A2*) of the 22 *UGT* genes have the optimal Kozak motif with an A or G at −3 and a G at +4 (Figure 6). In this study, we found 99 mutations in the 5′UTRs of *UGT* genes, 18 of which were located within the Kozak sequence with potential influence on translation initiation (Figure 6, Table 2, Table S6). For example, the conserved G at +4 in *UGT2B28* was mutated to T. This might reduce translation efficiency (Figure 6). Although the Kozak sequence does not generally extend beyond G in position +4, a C at position +5 is highly conserved in eukaryotic genes [88]. Consistent with this, 17 of the 22 *UGT* genes have a C at position +5 (Figure 6). Furthermore, the presence of a U at position +5 was shown to negate the effect of G at position +4 [86]. Therefore, mutations that change a U at position +5 to any of the three other bases may enhance translation efficiency. The Kozak sequences of two *UGT* genes (*UGT3A1*, *UGT2A1*) have a G at position +4 and a U at position +5, indicative of a weak element (Figure 6). We found a mutation within the *UGT3A1* Kozak sequence that changed the U to C at position +5, thus possibly enhancing translation efficiency (Figure 6). Finally, several *UGT* genes had mutations at positions −10 (*1A1*), +7 (*1A4*, *2B*4, *2B17*), +8 (*2B7*), +9 (*2B4*, *2B7*, *2B15*, *2B17*, *2B28*) and +10 (*1A5*, *2B10*) that are not within but adjacent/close to the Kozak sequence (Figure 6). As previously reported [85,86,87], these mutations likely have no or weak effects on translation initiation.

#### 3.2.8. Mutations in the 3′ UTRs of UGT Genes

**Figure 7:**
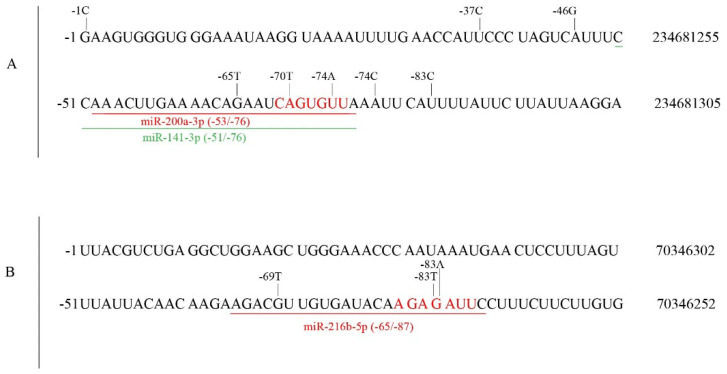
Somatic mutations within known miRNA seed target sites in UGT 3′UTRs in TCGA tumors. Data shown are the first 100 nt sequence of the 3′ UTR region for UGT1A (**A**) or UGT2B4 (**B**) with positions indicated at the cDNA (left, positioned as −1 for the nucleotide immediately following the stop codon) and genomic (GRCh37/hg19) (right) levels. Somatic mutations within miRNA target sites are given above the sequences. Underlined sequences are known target sites for miR-200a-3p (RED) and miR-141-3p (GREEN) in the UGT1A 3′UTR (**A**) and miR-216-5p (RED) in the UGT2B4 3′UTR (**B**). The miRNA positions are given in BRACKETs, and the miRNA seed target sites are highlighted in RED (**A**,**B**).

Fourteen of the 22 UGT mRNAs are known to be regulated by at least one miRNA via binding to their 3′UTRs [89]. In this study, we found 182 somatic mutations in the UGT 3′UTRs in TCGA tumors (Table 2, Table S7). Mutations within known miRNA target sites may affect miRNA regulation. Examples of such mutations include (1) two mutations in the UGT1A 3′UTR (*70A > T, *74T > A) within the seed target site that is shared by miRNA-200a-3p and miR-141-3p (Figure 7A) and (2) two mutations in the UGT2B4 3′UTR (*83G > T, *83G > A) within the miR-216b-5p seed target site (Figure 7B). As miRNAs regulate target mRNAs primarily via the binding of its seed to the seed target site [89], these mutations are likely to disrupt this binding with a significant impact on miRNA regulation. Furthermore, the pairing of the 3′ sequence of the miRNA to the 5′ sequence of the target site (3′ pairing) can also facilitate miRNA regulation [88]. We found many mutations that are located outside seed target sites but within the 5′ sequences of known miRNA target sites in the UGT2B7 (Figure S23A) and UGT2B15 (Figure S23B) 3′UTRs. The potential impact of these mutations on miRNA regulation remains to be investigated.

#### 3.2.9. Mutations in the Splice Sites of *UGT* Genes

Most canonical exons of human genes have a conserved acceptor splice site with the dinucleotide “AG” at the 5′-end and a conserved donor splice site with the dinucleotide “GT” at the 3′-end; therefore, mutations in splice sites, especially those within the dinucleotides AG and GT, can disrupt pre-mRNA splicing, leading to exon skipping or intron inclusion [90]. In this study, we found 26 mutations in the donor splice sites and 19 mutations in the acceptor splice sites of 12 *UGT* genes (*1A10*, *2A1*, *2A2*, *2A3*, *2B4*, *2B7*, *2B10*, *2B11*, *2B15*, *2B28*, *3A1* and *3A2*) in TCGA tumors (Table 2, Table S4). Of these 45 mutations, 38 occurred at the G base within the AG or GT dinucleotide, which was mutated to A (25 mutations), T (7 mutations) or C (4 mutations). Therefore, these mutations abolished the conserved dinucleotide AG or GT of splice sites and likely disrupted the splicing of the relevant exons (Table S4). 

#### 3.2.10. Recurrent Mutations in *UGT* Genes

We found 215 recurrent mutations with a total number of 519 mutations in *UGT* genes in TCGA tumors (Table S8). Table S8 lists the recurrent mutations for each of the 22 *UGT* genes, including 163, 31, 11, 6, 3 and 1 recurrent mutations that occurred 2, 3, 4, 5, 6 and 8 times, respectively. Nearly 80% (171/215) were missense, nonsense and small indels that result in amino acid substitutions, premature stop codons and frame-shift truncated proteins, respectively. Three *UGT* genes (*2B4*, *3A2* and *2B10*) had the largest numbers of recurrent mutations (29, 20 and 15, respectively). Recurrent mutations generally occurred in more than one cancer type. For example, the “Frame_Shift_Del” mutation [c.517delT(Trp173GlyfsTer8)] in *UGT1A4* exon 1 was observed in three different types of cancers, including one COAD tumor, two UCEC tumors and five STAD tumors (Table S4). Another “Frame_Shift_Del” mutation [1566delA (Arg524GlufsTer22)] in *UGT1A* exon 5 was also seen in three different types of cancers, including one BRCA tumor, three COAD tumors and two STAD tumors (Table S4). In contrast, some recurrent mutations were restricted to a specific cancer type. For example, the Frame_Shift_Del [c.364delT (Ser122GlnfsTer12)] was only seen in three UCEC tumors; the missense mutation [c.463C > T (Pro155Ser)] occurred only in five SKCM tumors (Table S4). 

### 3.3. Assesment of Associations of UGT Mutations with Clinicopathological Parameters Using the LUAD Cohort

**Figure 8:**
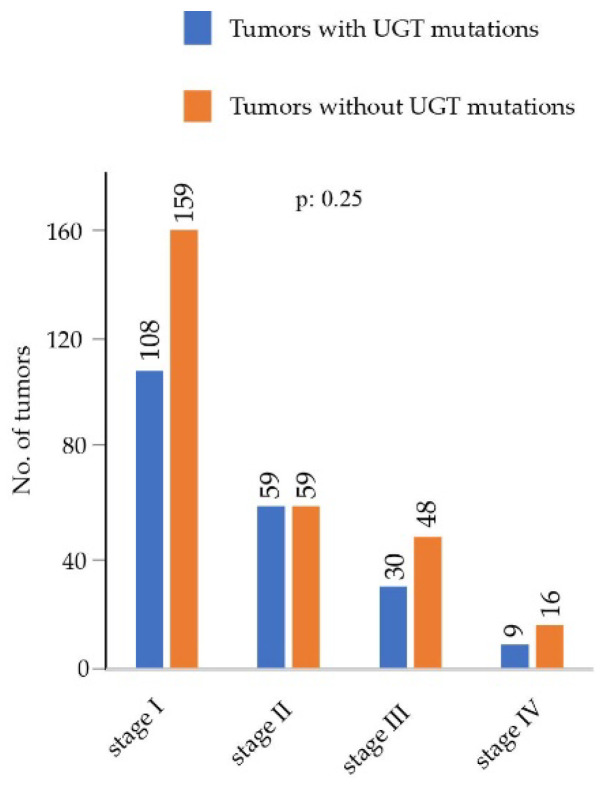
Assessment of potential associations of *UGT* mutations with tumor stages in the LUAD cohort. A graph shows the numbers of tumors at four different tumor stages in the LUAD cohort. Chi-squared tests. A *p* value of < 0.05 was considered statistically significant.

The LUAD cohort had 512 tumors with a total number of 243,687 somatic mutations, of which 235 tumors have *UGT* mutations with a total number of 412 somatic mutations in *UGT* genes (Table 1). The LUAD cohort represents a good model cancer type to assess whether *UGT* mutations are associated with clinicopathological parameters. All raw data used for analysis in this section are provided in Table S9. We obtained clinicopathological parameters such as tumor stages for 488 tumors and overall survival (OS) times for 495 patients from the TCGA Pan-Cancer Clinical Data Resources (TCGA-CDR), as we recently reported [39]. Figure 8 shows the numbers of tumors with or without UGT mutations at four different tumor stages (I, II, II and IV). Chi-squared tests showed that there was no significant difference in the mutation frequency of *UGT* genes across different stages (*p* = 0.25) (Figure 8). This indicates that mutations in *UGT* genes were not related to tumor stage.

We recently reported highly variable expression of *UGT* genes in LUAD tumors and a lack of expression of one or multiple *UGT* genes in many LUAD tumors [39]. Mutations in *UGT* genes might have an impact on clinicopathological parameters only if they occur in tumors that express the corresponding *UGT* gene. We focused on the analysis of 165 LUAD tumors with a total number of 267 mutations in *UGT* genes, such as missense, nonsense or small indels that are predicted to encode mutated proteins. We obtained the expression levels (RSEM) of all *UGT* genes in these tumors, as recently reported [39]. We found that 31% of *UGT* mutations (83/267) occurred in tumors that were previously classified to have a high expression of the corresponding *UGT* genes [39] (Table S9). However, this analysis also revealed differences in the overall levels of UGT expression between mutated and unmutated tumor groups (Table S9), suggesting that any comparison of clinicopathological features and clinical outcomes (e.g., survival time) between these groups could be confounded by differing UGT expression levels. For this reason, we did not attempt to perform survival analyses comparing the mutated and non-mutated cohorts within any cancer type.

### 3.4. Mutations in UGT Genes in Human Cancer Cell Lines

After having characterized the mutations of *UGT* genes in human cancers, we assessed the mutation profiles of *UGT* genes in 1568 CCLE cell lines. Overall, we found 895 mutations in *UGT* genes in 502 CCLE cell lines (Table 3, Table S5). Table 3 lists the number of mutations for each of the eight different types of mutations found in CCLE cell lines: (1) translation_start_site, (2) missense, (3) nonsense, (4) frame_shift_del, (5) frame_shift_ins, (6) in_frame_del, (7) nonstop and (8) splice site. Of the 895 mutations, 728 (81%) were missense mutations, 337 (45%) of which were SIFT-defined as deleterious amino acid substitutions with a significant impact on protein function.

Of the 502 CCLE cell lines with mutations in *UGT* genes, 172 had multiple mutations in *UGT* genes (Table S5). Briefly, there were 89, 48, 15, 5, 4 and 4 cell lines that had 2, 3, 4, 5, 6 and 7 mutations in *UGT* genes, respectively. Seven other cell lines that were possibly derived from hypermutated tumors had 10 (COLO792), 11 (HCC2998, SNU1040), 12 (GP5D, MEWO, SNU81) or 15 (SW684) mutations in *UGT* genes. Multiple mutations within a single cell line were usually distributed across several *UGT* genes, although some cell lines showed mutations clustered in a single *UGT* gene (Table S5). For example, the prostate cancer DU145 cell line had two mutations in *UGT1A10* [c.13G > T (Gly5Trp); c.13_14delinsTT (Gly5Val)] and one mutation in four other *UGT* genes, including *UGT2B4* [c.1372G > T (Asp458Tyr)], *UGT2B15* [c.728G > C (Arg243Thr)], *UGT2B17* [c.32T > A (Leu11Gln)] and *UGT2B28* [c.1262C > A (Ser421Ter)]. In contrast, the breast cancer ZR751 cell line had three mutations in *UGT2B28* [c.357_358delinsAT (Phe119_His120delinsLeuTyr); c.357T > A (Phe119Leu); c.358C > T (His120Tyr)]. 

Of the 895 mutations in *UGT* genes in CCLE cell lines, 150 were recurrent mutations (Table S5). There were 48, 11, 1, 1 and 2 mutations in *UGT* genes that reoccurred in 2, 3, 4, 5 and 6 different CCLE cell lines, respectively. The majority of CCLE cell lines with the same recurrent mutations in *UGT* genes were derived from different types of tumors. For example, the mutation c.518T > G (Leu173Arg) in *UGT1A9* was observed in five CCLE cell lines (JHOM1, LUDLU1, CAPAN1, TCCSUP and HT29) that were derived from five different types of cancers (ovary cancer, non-small cell lung cancer, pancreatic cancer, bladder cancer and colorectal cancer, respectively) (Table S5). 

A comparison of the 3427 mutations in TCGA tumors and the 895 mutations in CCLE cell lines identified 114 mutations in *UGT* genes that were found in both TCGA tumors and CCLE cell lines (Table 2, Table 3 and Table S10). Nearly one-third of these mutations were present in CCLE cell lines derived from the same types of tumors that shared the mutations, suggesting that they might be derived from the parental tumors. Table S10 shows the number of mutations for every *UGT* gene that occurred in both TCGA tumors and CCLE cell lines. For example, 10 of the 36 mutations in *UGT1A7* in the CCLE cell lines were also found in TCGA tumors (Table S10). 

## 4. Discussion

We recently showed abundant expression of *UGT* genes in human cancers and their association with clinical outcomes, highlighting the importance of the intratumoral metabolism of drugs and pro/anti-cancer signaling molecules through the UGT conjugation pathway [39]. Somatic mutations in *UGT* genes in the tumor that could influence this pathway have not yet been reported. In the present study, our assessment of the mutation profiles of 1069 tumors from 33 TCGA cancer types revealed for the first time the somatic mutation landscape for all 22 *UGT* genes in human cancers. Briefly, nearly one-fifth of the tumors analyzed had mutations in *UGT* genes with a total number of 3427 somatic mutations. Most mutations occurred sporadically throughout the coding sequences of *UGT* genes, but recurrent mutations and mutation hotspot regions were also observed. The impact of mutations on protein function depends on the position and type of mutation. Approximately two-thirds of the mutations in *UGT* genes in tumors were missense, frame-shift and nonsense mutations that may directly affect UGT function via coding for variant or truncated proteins. However, this direct impact may only occur in the tumors that express the mutated *UGT* proteins. Our analysis of the LUAD tumors indicates that approximately 31% of these mutations occurred in the tumors that expressed the corresponding *UGT* genes. Mutations in non-coding regions do not alter protein sequence but may indirectly influence UGT function through modulating the efficiency of translation initiation (mutations within the Kozak sequence in 5′ UTRs), disrupting miRNA regulation (mutations in 3′ UTRs) or altering pre-mRNA splicing process (mutations in splice sites). Collectively, somatic mutations occurred throughout the exonic sequences of *UGT* genes with a potential impact on local UGT activity within the tumor through multiple mechanisms.

Cancer genomes acquire somatic mutations during cancer development and progression that are generally classified into driver and passenger mutations [91,92,93,94]. Driver mutations contribute to cancer initiation or promote tumor growth; passenger mutations accumulate through tumor evolution with no or even detrimental effects on tumor growth [93,95]. On average, every cancer genome has 4–5 driver mutations, with the vast majority of mutations being passenger mutations [96]. Genes with driver mutations in at least one cancer type are considered to be cancer driver genes [93,97]. A recent PanCancer and PanSoftware analysis of the MC3 somatic mutations in 9423 tumors from 33 TCGA cancer types identified 299 cancer driver genes [52]. *UGT* genes were not among these cancer driver genes, and driver mutations in *UGT* genes have not yet been reported in other similar studies [52,96,98]. However, more than half of our observed somatic mutations in *UGT* genes are predicted to code for truncated proteins or variant proteins with deleterious amino acid substitutions. Given that UGT proteins dimerize, and truncated inactive forms have been shown to act in a dominant-negative manner, it is possible for even heterozygous mutations of *UGT* genes to lead to a significant loss of UGT function [82,83,84]. Our findings therefore support the potential role of UGT somatic mutations in modulating cancer growth and treatment, as described in detail below.

Numerous drugs and their active metabolites are UGT substrates, including chemotherapy drugs such as etoposide, epirubicin and irinotecan [5,16,99,100]. Mutations in *UGT* genes in the tumor that reduce the glucuronidation of anticancer drugs can increase intratumoral drug concentrations, thus potentially enhancing therapy efficacy and inhibiting tumor growth. For example, irinotecan is commonly used for treating colorectal cancer (COAD), and its active metabolite, SN-38, is primarily glucuronidated by UGT1A1 with weak activity from all other UGT1As except UGT1A4 [101,102,103,104,105]. We recently showed the expression of all nine UGT1As at varying levels in COAD tumors, suggesting that there is in situ glucuronidation of SN-38 within the tumor [39]. In the present study, we found seven COAD tumors with a frame-shift [c.1566delA (Arg524GlufsTer22)] or deleterious amino acid substitution mutation in the shared *UGT1A* exons 2–5 [c.959C > A (Ala320Asp), c.983A > C (Gln328Pro), c.1145G > A (Gly382Asp), c.1474G > A (Val492Met)]. These mutations affect all nine UGT1A enzymes and could abolish or significantly reduce intratumoral glucuronidation of SN-38, thus increasing local drug concentration and efficacy. Similar mutations in *UGT1A* exons 2–5 were also frequently seen in six other cancer types (i.e., BRCA, LUAD, LUSC, SKCM, STAD and UCEC) (Table S4). Treatments with irinotecan have been reported for all these six cancer types, suggesting that somatic mutations in *UGT1A* exons 2–5 could modulate irinotecan efficacy in these cancers [106,107,108,109,110]. As mentioned earlier, germline genetic polymorphisms such as low activity *UGT1A1* alleles result in high systemic SN-38 levels due to reduced hepatic clearance and hence increase the risk of hematological and gastrointestinal toxicity following irinotecan administration [18]. This raises the possibility of genotyping tumors for deleterious somatic *UGT1A* mutations as a strategy to identify patients that may show greater irinotecan efficacy, including at lower doses that reduce the risk of toxicity. 

Another example of *UGT* somatic mutations that may have an impact on drug responses relates to the treatment of estrogen receptor-positive breast cancers with antiestrogens such as tamoxifen (TAM) and aromatase inhibitors such as exemestane (EXE) [111]. The active metabolites of TAM and EXE are 4-OH-TAM and 17-OH-EXE, which are glucuronidated by UGT2B15 and UGT2B17, respectively [112,113]. We found BRCA tumors with deleterious somatic mutations in *UGT2B15* and *UGT2B17* (Table S4). It is anticipated that these mutations could decrease the glucuronidation of 4-OH-TAM or 17-OH-EXE within the tumor, potentially enhancing drug efficacy and inhibiting breast tumor growth. 

In addition to drugs, numerous endogenous (e.g., fatty acids, bile acids, bilirubin and steroid hormones) and exogenous (e.g., dietary constituents, carcinogens) pro/anti-cancer molecules are UGT substrates [5]. For example, estrogens contribute to breast carcinogenesis and promote breast cancer growth [114]. Six UGTs (1A1, 1A3, 1A8, 1A9, 1A10, 2B7) that are expressed in breast cancer have been shown to conjugate estrogens, such as estrone (E1) and 17β-estradiol (E2) [39,115]. In the present study, we found four BRCA tumors with a frame-shift [c.1566delA (Arg524GlufsTer22)] or deleterious amino acid substitution mutation [c.1174G > T (Gly392Cys), c.1175G > T (Gly392Val), c.1326G > A (Met442Ile)] within the shared *UGT1A* exons 2–5. A further 14 BRCA tumors had similar mutations that specifically affect one of the aforementioned six UGT1As (Table S4). It is anticipated that these mutations could reduce the glucuronidation of estrogens within the tumor, thus increasing local estrogen levels and stimulating tumor growth. As mentioned earlier, androgens are implicated in prostate carcinogenesis and promote androgen-sensitive prostate cancer growth. Androgens are primarily inactivated in the prostate by UGT2B15 and UGT2B17 [25,26]. In the present study, we found two mutations in *UGT2B15* [c.249A > T (Lys83Asp), c.436T > A (Phe146Ile)] and one mutation in *UGT2B17* [c.1193C > G (Ala398Gly)] in PRAD tumors (Table S3). It is anticipated that these somatic mutations could reduce androgen inactivation, thus potentially promoting androgen-sensitive prostate cancer growth.

The impact on the intratumoral metabolism of drugs and pro/anti-cancer signaling molecules is likely to be more profound in tumors with multiple mutations in *UGT* genes. As described earlier, four cancers with a high mutation burden (SKCM-metastatic, SKCM-primary, LUAD and UCEC) had the largest percentages (46%, 29%, 19% and 10%, respectively) of tumors with two or more mutations from *UGT* genes. This is particularly true for hypermutated tumors. Among the 53 hypermutated tumors that were excluded from analysis in this study, forty-six had ten or more mutations from *UGT* genes (Table S1). Our preliminary analysis of the MC3 MAF file revealed widespread mutations in other drug-metabolizing enzymes (e.g., CYPs, SULTs and GSTs) and ABC and SLC transporters in TCGA tumors. It is anticipated that multiple concurrent mutations that simultaneously affect UGTs and other drug-metabolizing enzymes and transporters would have a great impact on the capacity of cancer cells to uptake, metabolize and dispose of anticancer drugs and other pro/anti-cancer molecules. 

Human cancer cell lines have been used for experimental models for the study of cancer biology and therapy for decades [53,54]. Cancer cell lines are also used to study the function and regulation of *UGT* genes [3,5,89]. Our finding of mutations in *UGT* genes in over 500 cancer cell lines emphasizes the importance of the selection of proper cell lines that have no mutations in the *UGT* genes under investigation. For example, *UGT2B28* is highly upregulated upon androgen exposure in the ZR751 breast cancer cell line, which would suggest that it is a suitable line to study the biological function of this gene in breast cancer [116]. However, as described earlier, the presence of three deleterious mutations in *UGT2B28* in the ZR751 cell line indicates that it is unsuitable for functional studies of this gene. 

One limitation of the present study is that the MC3 data derive mainly from primary, treatment-naïve tumors. It is likely that drug treatment results in selective pressure that enriches mutations that provide a growth/survival advantage. It is possible that pre- and post-treatment tumors have different profiles of mutations in *UGTs* that control intratumoral drug exposure; however, the analysis of such paired pre- and post-treatment datasets is a subject for future study. Another notable consideration is that the levels of UGT expression may vary in tumors with and without UGT mutations. Mutational status and expression level are often treated as independent variables in analyses of clinicopathological features and clinical outcomes. However, we found that the overall expression levels of UGTs were not comparable between the mutated and unmutated groups within the LUAD cohort, suggesting that such analyses should be treated with caution. Future studies could develop approaches to integrate these variables in analyses of clinical outcomes.

## 5. Conclusions

In conclusion, our comprehensive assessment of the mutation profiles in 1069 TCGA tumors and 1568 CCLE cell lines identified 3427 and 895 mutations in *UGT* genes in human cancers and cancer cell lines, respectively. Over half of the mutations in *UGT* genes in tumors are predicted to encode truncated proteins or variant proteins with deleterious amino acid substitutions that likely influence the capacity of cancer cells to metabolize anticancer drugs and pro/anti-cancer signaling molecules through the UGT conjugation pathway. As a result, somatic mutations in *UGT* genes might affect tumor growth and therapeutic efficacy, suggesting their potential role as biomarkers predicting therapeutic efficacy and clinical outcomes. We acknowledge the necessity for future experimental and prospective clinical studies to further validate this hypothesis. Overall, we consider this study an important first step in identifying the mutational profiles of *UGTs* and other genes associated with drug metabolism and disposition in tumors, which could ultimately aid in the development of personalized cancer therapies. 

## Figures and Tables

**Table 1:** Somatic mutations in *UGT* genes in 33 different TCGA cancer types.

Cancer types	Description	No. of Tumors	Total No. of Mutations	No. of Mutations per Tumor	No. of Tumors with UGT Mutations	Total No. of UGT Mutations	No. of Missense Frameshift Nonsense	Percentage of Tumors with UGT Mutations
ACC	Adrenocortical Carcinoma	92	11,981	130	9	14	8	0.097
BLCA	Bladder Urothelial Carcinoma	411	155,233	377	102	145	90	0.248
BRCA	Breast Invasive Carcinoma	1019	135,026	132	86	125	78	0.084
CESC	Cervical Squamous Cell Carcinoma and							
	Endocervical Adenocarcinoma	289	83,232	288	48	71	47	0.168
CHOL	Cholangiocarcinoma	36	4500	125	3	3	3	0.083
COAD	Colon Adenocarcinoma	402	242,404	602	102	218	159	0.253
DLBC	Lymphoid Neoplasm Diffuse Large							
	B-cell Lymphoma	37	7,784	210	5	5	3	0.142
ESCA	Esophageal Carcinoma	183	48,196	263	27	31	20	0.147
GBM	Glioblastoma Multiforme	380	40,617	106	82	96	66	0.215
HNSC	Head and Neck Squamous Cell Carcinoma	507	125,417	247	106	147	83	0.209
KICH	Kidney Chromophobe	66	3324	50	4	4	3	0.060
KIRC	Kidney Renal Clear Cell Carcinoma	371	32,001	86	25	28	20	0.067
KIRP	Kidney Renal Papillary Cell Carcinoma	281	35,445	126	32	33	24	0.113
LAML	Acute Myeloid Leukemia	140	8332	59	6	10	5	0.042
LGG	Brain Lower Grade Glioma	512	24,000	46	34	37	30	0.066
LIHC	Liver Hepatocellular Carcinoma	363	60,432	166	45	50	32	0.123
LUAD	Lung Adenocarcinoma	512	243,687	475	235	412	287	0.458
LUSC	Lung Squamous Cell Carcinoma	484	204,623	422	166	253	182	0.342
MESO	Mesothelioma	82	3979	48	1	1	1	0.012
OV	Ovary Serous Cystadenocarcinoma	406	53,115	130	60	71	44	0.147
PCPG	Pheochromocytoma and Paraganglioma	179	2726	15	4	4	1	0.022
PAAD	Pancreatic Adenocarcinoma	155	8728	56	3	3	2	0.019
PRAD	Prostate Adenocarcinoma	495	24,778	50	19	22	15	0.038
READ	Rectum Adenocarcinoma	146	30,380	208	19	26	18	0.13
SARC	Sarcoma	236	31,678	134	33	41	26	0.139
SKCM	Skin Cutaneous Melanoma (primary)	103	68,991	669	56	160	94	0.543
SKCM	Skin Cutaneous Melanoma (Metastatic)	364	438,405	1204	246	926	592	0.675
STAD	Stomach Adenocarcinoma	437	221,714	507	105	175	120	0.240
TGCT	Testicular Germ Cell Tumor	144	3588	24	0	0	0	0.000
THCA	Thyroid Carcinoma	491	12,050	24	9	10	8	0.018
THYM	Thymoma	122	4888	40	2	3	2	0.016
UCEC	Uterine Corpus Endometrial Carcinoma	487	307,642	631	122	297	193	0.250
UCS	Uterine Carcinoma	57	5261	92	5	5	3	0.087
UVM	Uveal Melanoma	80	1935	24	1	1	1	0.012
**SUM**		**10,069**	**2,686,092**	**266**	**1802**	**3427**	**2260**	**0.178**

**Table 2:** Somatic mutations for each of the 22 *UGT* genes in TCGA cancers.

UGT Gene	5′UTR	Missense	Translation Start Site	Nonsense	Silent	Frame Shift Del	Frame Shift Ins	In Frame Del	In Frame Ins	Non Stop	Intron	Splice Site	3′UTR	Sum	RefSeq Transcript
1A8	3	47 (26)		8	19	4	2							83	ENST00000373450
1A10	3	59 (31)		3	24	4						1		94	ENST00000344644
1A9	2	56 (27)		7	19	5	7							96	ENST00000354728
1A7		46 (25)		3	12	4	3							68	ENST00000373426
1A6	14	43 (18)	1	5	19	1								83	ENST00000305139
1A5		48 (18)		2	24	1	1							76	ENST00000373414
1A4	1	49 (17)		4	20	10	2							86	ENST00000373409
1A3	1	47 (18)		2	24	4	1							79	ENST00000482026
1A1	2	42 *			32	2	1							79	ENST00000609767
1A E2–5		43 (29)		1	25	7		2				1	8	87	ENST00000344644 ENST00000373409
2A1	5	44 (29)		5	15	3		1			2	3		78	ENST00000514019
2A2		49 (15)		7	15	2								73	ENST00000457664
2A1/2A2 E2–6		70 (53)		11	22	6					1	5	6	121	ENST00000514019 ENST00000503640
2A3	2	127 (84)		10	33	7	3					3	11	196	ENST00000251566
2B4	7	151 (93)		21	74	5	1	2				6	22	289	ENST00000305107
2B7	2	106 (65)		4	37	4					1	3	15	172	ENST00000305231
2B10	3	116 (77)		11	27	4	2				3	7	20	193	ENST00000265403
2B11	20	115 (79)		16	38	3					1	4	8	205	ENST00000446444
2B15	2	122 (78)		14	38	3	1			1	1	1	27	210	ENST00000338206
2B17	1	100 (61)		10	29	5	2							147	ENST00000317746
2B28	1	133 (80)		15	47	1						4	9	210	ENST00000335568
3A1	20	153 (64)		9	68	2	1				19	6	29	307	ENST00000274278
3A2	10	143 (65)		12	64	3	1					1	21	255	ENST00000282507
UGT8		89 (47)		4	29	3	1				8		6	140	ENST00000310836
**Total**	**99**	**1998 (1099)**	**1**	**184**	**754**	**93**	**29**	**5**		**1**	**36**	**45**	**182**	**3427**	

1A E2–5: exons 2–5 shared by all nine *UGT1A*s. 2A1/2A2 E2–6: exons 2–6 shared by *UGT2A1* and *UGT2A2*. The number in the BRACKET refers to the number of deleterious missense mutations. *: none of these missense mutations were assessed by the SIFT algorithm in the MC3 MAF file.

**Table 3:** Mutations in *UGT* genes in 1568 CCLE (Cancer Cell Line Encyclopedia, Broad 2019) cell lines.

UGT Genes	CCLE Cell Lines with UGT Mutations	Translation Start Site	Missense	Nonsense	Frame Shift Del	Frame Shift Ins	In Frame Del	Nonstop	Splice Site	SUM
1A8	28		20	2	8	4				34 (5)
1A10	35		36	2						38 (1)
1A9	31		28		4	6				39 (4)
1A7	35	1	32	1	2	1				36 (10)
1A6	16		12	1	2	1				16 (3)
1A5	17		13		1	2	1			17 (2)
1A4	30		21		7	2			1	31 (2)
1A3	21		11		6	3	1			21
1A1	14		15							15 (1)
1A E2–5	22		19		6	1				26 (6)
2A1	15		13		2					15
2A2	22		22							22 (2)
2A1/2A2 E2–6	30		13	3					3	19 (1)
2A3	45		42	4	4	1				51 (7)
2B4	50		49	6	3	1				59 (10)
2B7	42		33	4	2	1			2	42 (6)
2B10	60		49	5	4	1			3	62 (11)
2B11	54		51	3					2	56 (7)
2B15	46		45	3		1				49 (8)
2B17	18		16	1	1				1	19 (1)
2B28	66		64	7	2	1			2	76 (10)
3A1	50		49	4	2				3	58 (6)
3A2	55	1	47	3	6	1		2		60 (7)
UGT8	29		28	2	3	1				34 (4)
**Total**	**502**	**2**	**728**	**51**	**65**	**28**	**2**	**2**	**17**	**895 (114)**

1A E2–5: exons 2–5 shared by all nine *UGT1A*s. 2A1/2A2 E2–6: exons 2–6 shared by *UGT2A1* and *UGT2A2*. The number in the BRACKET refers to the number of mutations that were also seen in TCGA tumors.

## Data Availability

The MC3 MAF file (mc3.v0.2.8.PUBLIC.maf.gz) containing the somatic mutation profiles of TCGA tumors is available from the PanCanAtlas website (https://gdc.cancer.gov/about-data/publications/pancanatlas) (accessed on 1 August 2022). The mutation profiles from the CCLE cell lines are available from the DepMap portal (https://depmap.org/portal) (accessed on 1 August 2022) and the CbioPortal (https://www.cbioportal.org) (accessed on 1 August 2022).

## Supplementary Materials

The following supporting information can be downloaded at: https://www.mdpi.com/article/10.3390/cancers14225708/s1, Figure S1: Mutations within the *UGT1A1* exon 1 in TCGA tumors; Figure S2: Mutations within the *UGT1A3* exon 1 in TCGA tumors; Figure S3: Mutations within the *UGT1A4* exon 1 in TCGA tumors; Figure S4: Mutations within the *UGT1A5* exon 1 in TCGA tumors; Figure S5: Mutations within the *UGT1A6* exon 1 in TCGA tumors; Figure S6: Mutations within the *UGT1A7* exon 1 in TCGA tumors; Figure S7: Mutations within the *UGT1A8* exon 1 in TCGA tumors; Figure S8: Mutations within the *UGT1A9* exon 1 in TCGA tumors; Figure S9: Mutations within the *UGT1A10* exon 1 in TCGA tumors; Figure S10: Mutations within the *UGT2A1* exon 1 in TCGA tumors; Figure S11: Mutations within the *UGT2A2* exon 1 in TCGA tumors; Figure S12: Mutations at the *UGT2A3* gene in TCGA tumors; Figure S13: Mutations at the *UGT2B7* gene in TCGA tumors; Figure S14: Mutations at the *UGT2B10* gene in TCGA tumors; Figure S15: Mutations at the *UGT2B11* gene in TCGA tumors; Figure S16: Mutations at the *UGT2B15* gene in TCGA tumors; Figure S17: Mutations at the *UGT2B17* gene in TCGA tumors: Figure S18: Mutations at the *UGT2B28* gene in TCGA tumors; Figure S19: Mutations within the codons of conserved amino acids across the UGT1A and UGT2B family enzymes; Figure S20: Mutations at the *UGT3A1* gene in TCGA tumors; Figure S21: Mutations at the *UGT3A2* gene in TCGA tumors; Figure S22: Mutations at the *UGT8* gene in TCGA tumors; Figure S23: Functional miRNA target sites and somatic mutations at 3′ untranslated regions of UGT mRNAs in TCGA tumors; Table S1: The tumors for each of the 33 different types of TCGA cancers that were included in the analysis of this study; Table S2: The tumors for each of the 33 different types of TCGA cancers that had somatic mutations at *UGT* genes; Table S3: Somatic mutations at *UGT* genes for each of the 33 different types of TCGA cancers; Table S4: Somatic mutations in TCGA tumors for each of the 22 *UGT* genes; Table S5: The CCLE cell lines assessed by this study and the mutations found in these cell lines for each of the 22 *UGT* genes; Table S6: Mutations at 5′ untranslated regions (UTRs) of *UGT* genes in TCGA tumors; Table S7: Mutations at 3′ untranslated regions (UTRs) of *UGT* genes in TCGA tumors. Mutations in functional miRNA seed target sites are in bold; Table S8: Recurrent somatic mutations at *UGT* genes in TCGA tumors; Table S9: Assessment of associations of UGT mutations with clinicopathological parameters in the LUAD cohort; Table S10: Mutations of *UGT* genes found in both TCGA tumors and CCLE cell lines.
