# Supplementary material for: The Somatic Mutation Landscape of UDP-Glycosyltransferase (UGT) Genes in Human Cancers
Source: Cancers (Basel). 2022 Nov 21;14(22):5708. doi: 10.3390/cancers14225708 (PMC9688768; doi:10.3390/cancers14225708)
Supplement: Supplementary file 1 [file cancers-14-05708-s001.zip › Supplemental Figures.pdf]

Article

The Somatic Mutation Landscape of UDP-Glycosyltransferase (*UGT*) Genes in Human Cancers

Dong Gui Hu*, Shashikanth Marri, Julie-Ann Hulin, Ross A McKinnon, Peter I. Mackenzie, and Robyn Meech

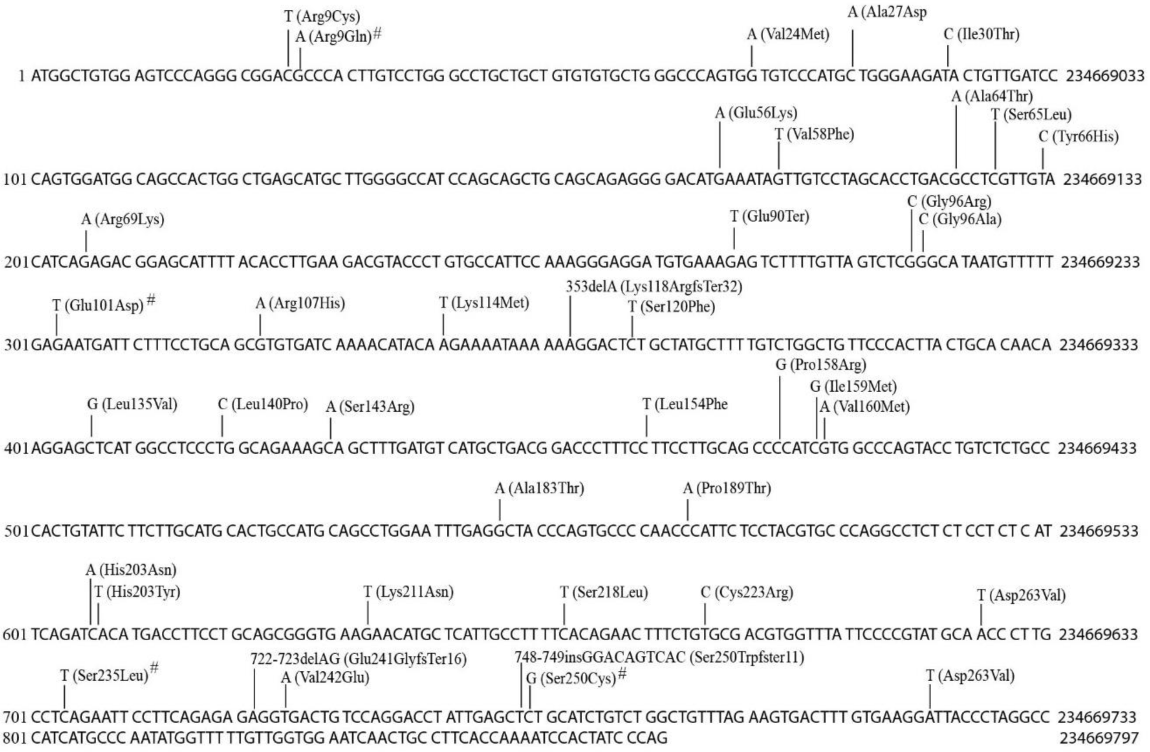

Figure S1. Mutations within the *UGT1A1* exon 1 in TCGA tumors. Data shown is the coding region of the *UGT1A1* exon 1 (864 bp) with genomic (GRCh37/hg19, right) and cDNA (NM_00463.3, left) positions. Mutations (missense, small indels) and the resulting changes at the protein level (NP_000454.1) are indicated above the reference mRNA sequence. Recurrent mutations are indicated by # (twice).

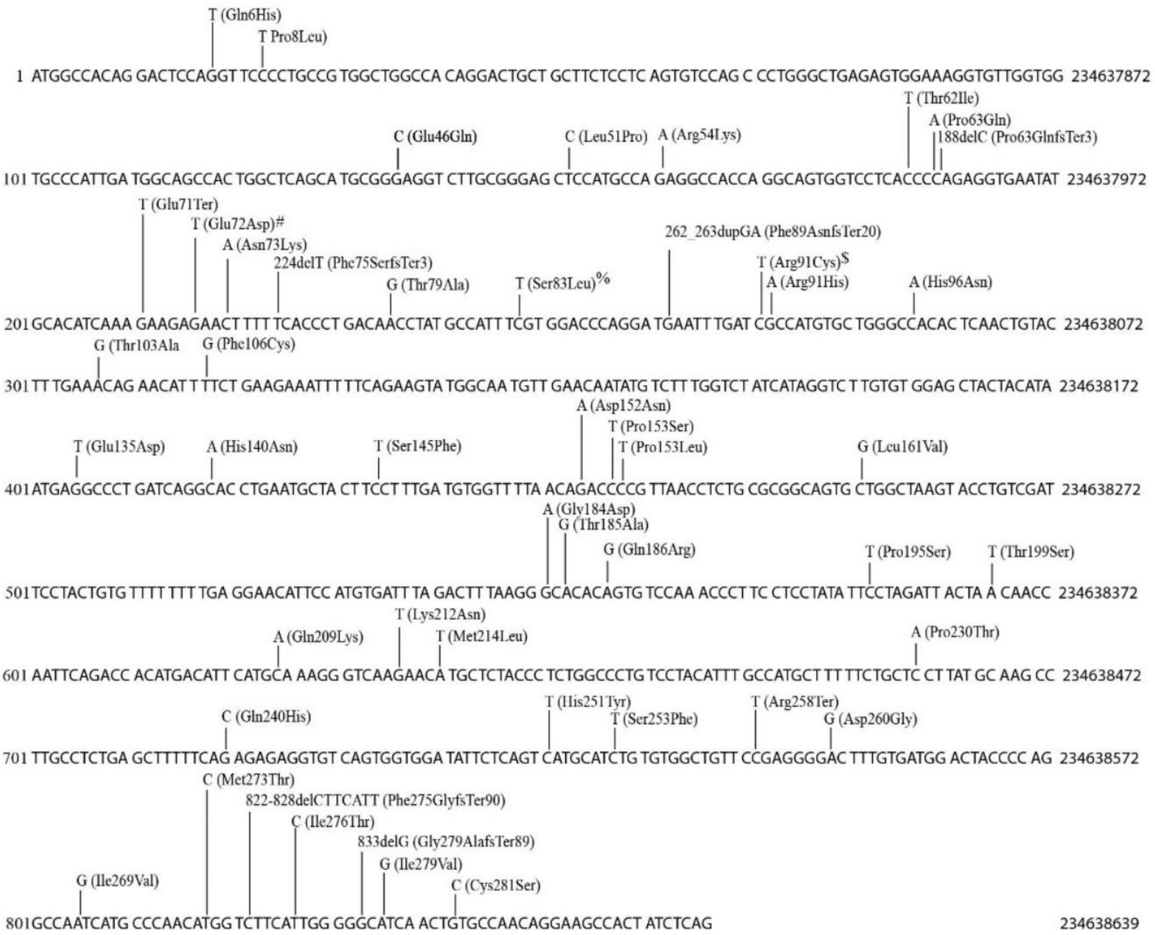

Figure S2. Mutations within the *UGT1A3* exon 1 in TCGA tumors. Data shown is the coding region of the *UGT1A3* exon 1 (867 bp) with genomic (GRCh37/hg19, right) and cDNA (NM_019093.4, left) positions. Mutations (missense, nonsense and small indels) and the resulting changes at the protein level (NP_061966.1) are indicated above the reference mRNA sequence. Recurrent mutations are indicated by # (twice), $ (three times), % (four times).

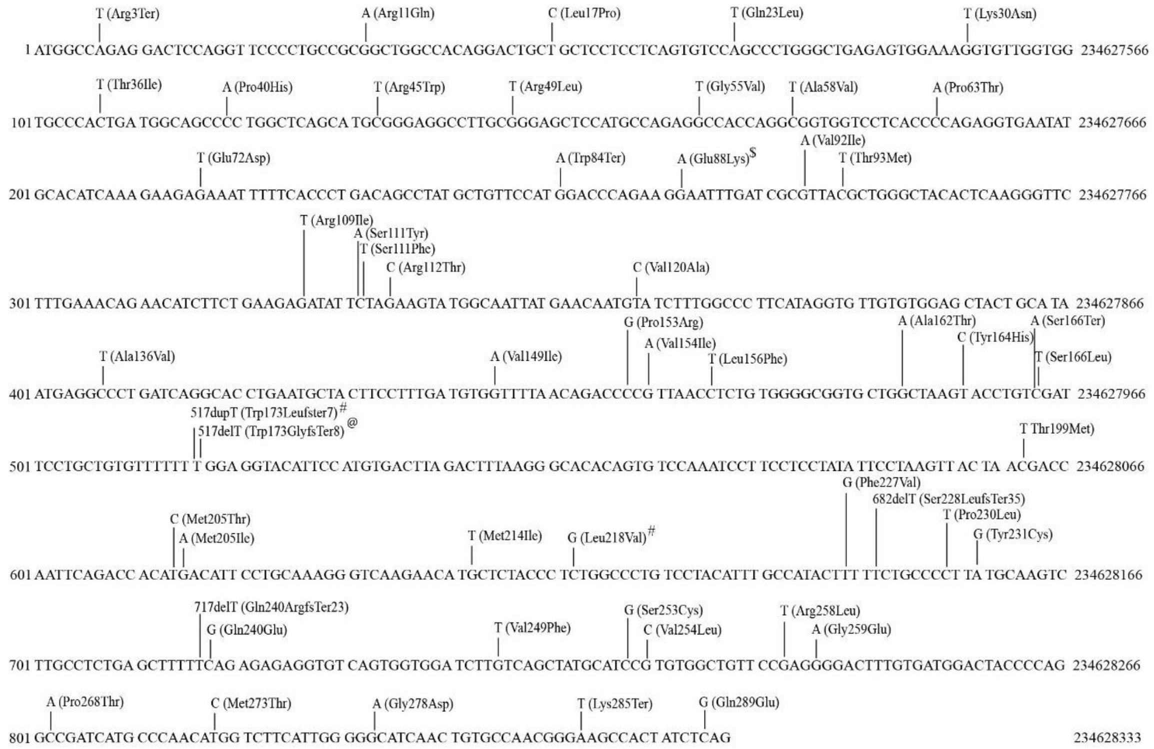

Figure S3. Mutations within the *UGT1A4* exon 1 in TCGA tumors. Data shown is the coding region of the *UGT1A4* exon 1 (867 bp) with genomic (GRCh37/hg19, right) and cDNA (NM_007120.3, left) positions. Mutations (missense, nonsense and small indels) and the resulting changes at the protein level (NP_009051.1) are indicated above the reference mRNA sequence. Recurrent mutations are indicated by # (twice), $ (three times), @ (eight times).

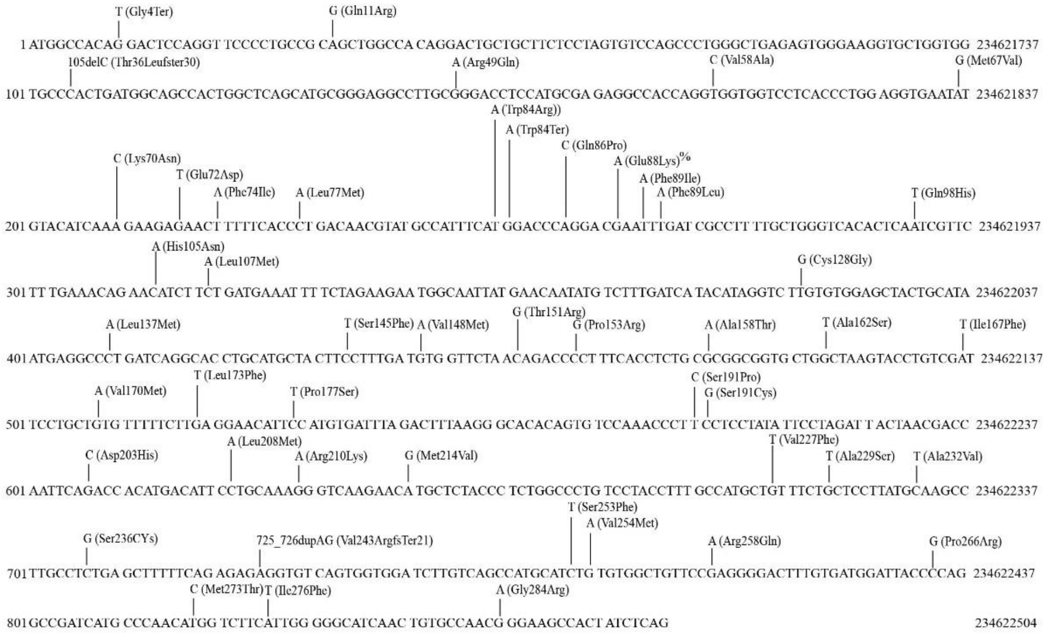

Figure S4. Mutations within the *UGT1A5* exon 1 in TCGA tumors. Data shown is the coding region of the *UGT1A5* exon 1 (867 bp) with genomic (GRCh37/hg19, right) and cDNA (NM_019078.2, left) positions. Mutations (missense, nonsense and small indels(and the resulting changes at the protein level (NP_061951.1) are indicated above the reference mRNA sequence. Recurrent mutations are indicated by % (four times).

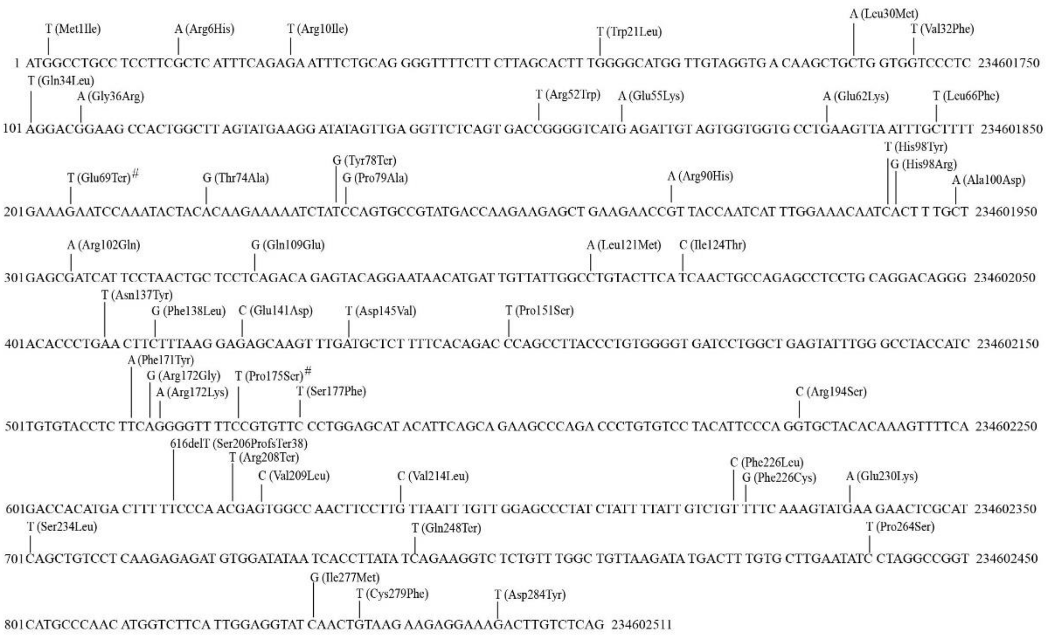

Figure S5. Mutations within the *UGT1A6* exon 1 in TCGA tumors. Data shown is the coding region of the *UGT1A6* exon 1 (861 bp) with genomic (GRCh37/hg19, right) and cDNA (NM_001072.4, left) positions. Mutations (missense, nonsense and small indels) and the resulting changes at the protein level (NP_001063.2) are indicated above the reference mRNA sequence. Recurrent mutations are indicated by # (twice).

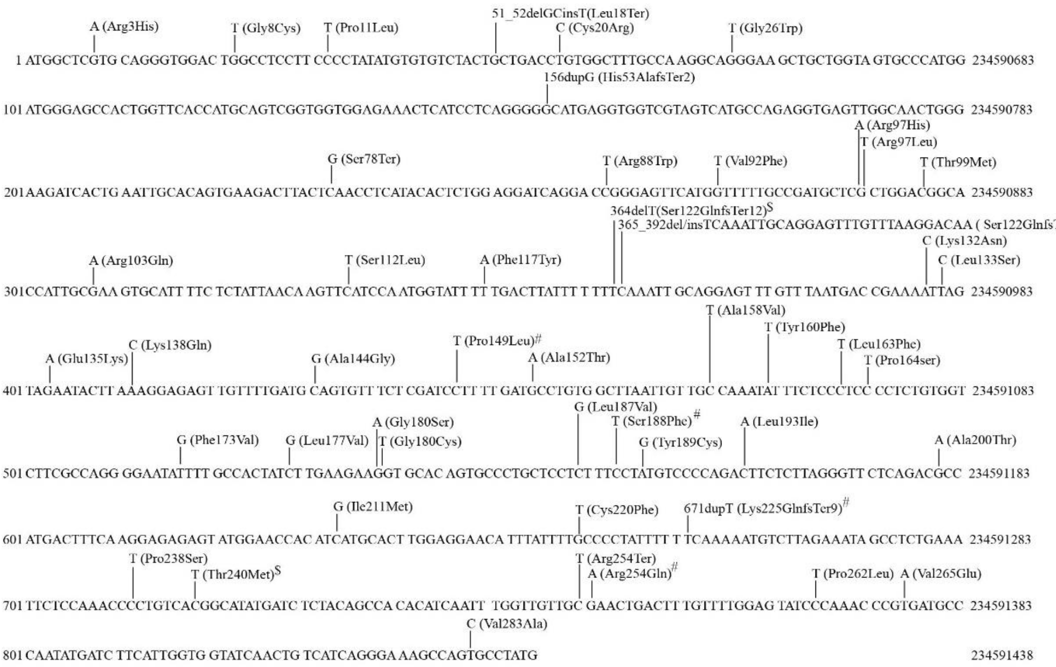

Figure S6. Mutations within the *UGT1A7* exon 1 in TCGA tumors. Data shown is the coding region of the *UGT1A7* exon 1 (855 bp) with genomic (GRCh37/hg19, right) and cDNA (NM_019077.3, left) positions. Mutations (missense, nonsense and small indels) and the resulting changes at the protein level (NP_061950.2) are indicated above the reference mRNA sequence. Recurrent mutations are indicated by # (twice), $ (three times).

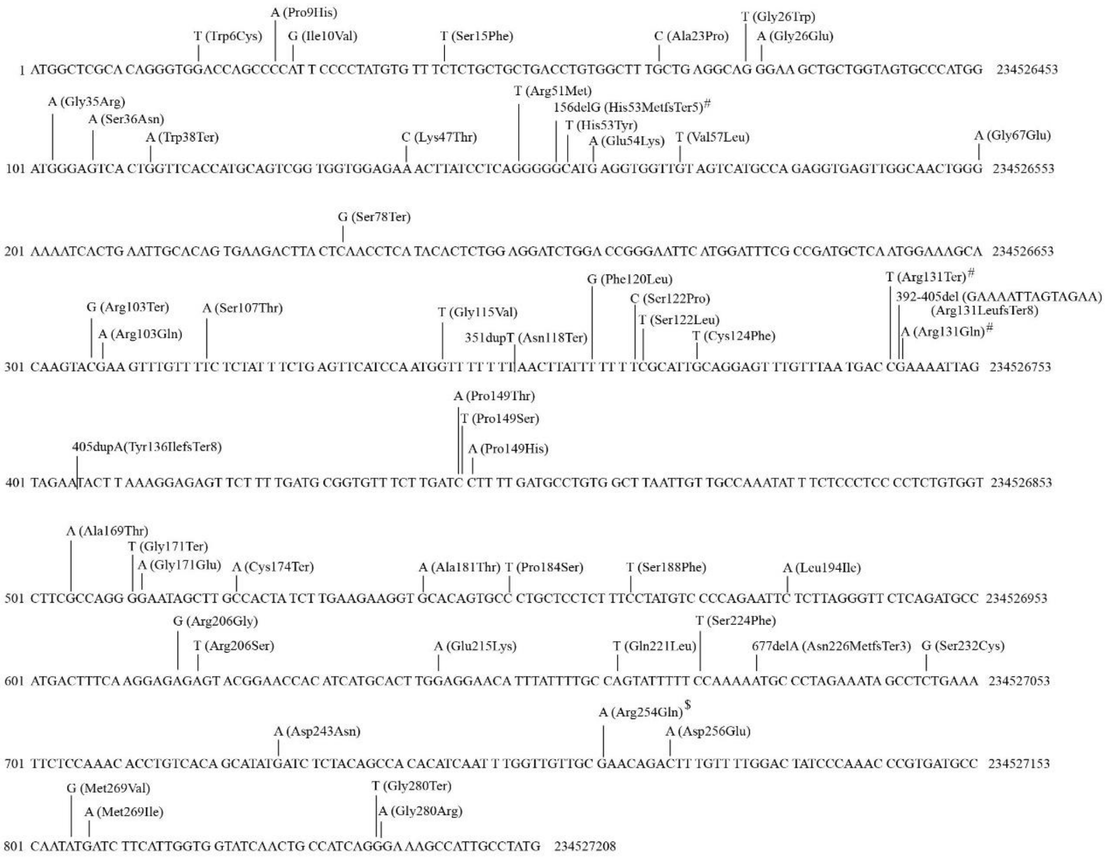

Figure S7. Mutations within the *UGT1A8* exon 1 in TCGA tumors. Data shown is the coding region of the *UGT1A8* exon 1 (855 bp) with genomic (GRCh37/hg19, right) and cDNA (NM_019076.5, left) positions. Mutations (missense, nonsense and small indels) and the resulting changes at the protein level (NP_061949.3) are indicated above the reference mRNA sequence. Recurrent mutations are indicated by # (twice), $ (three times).

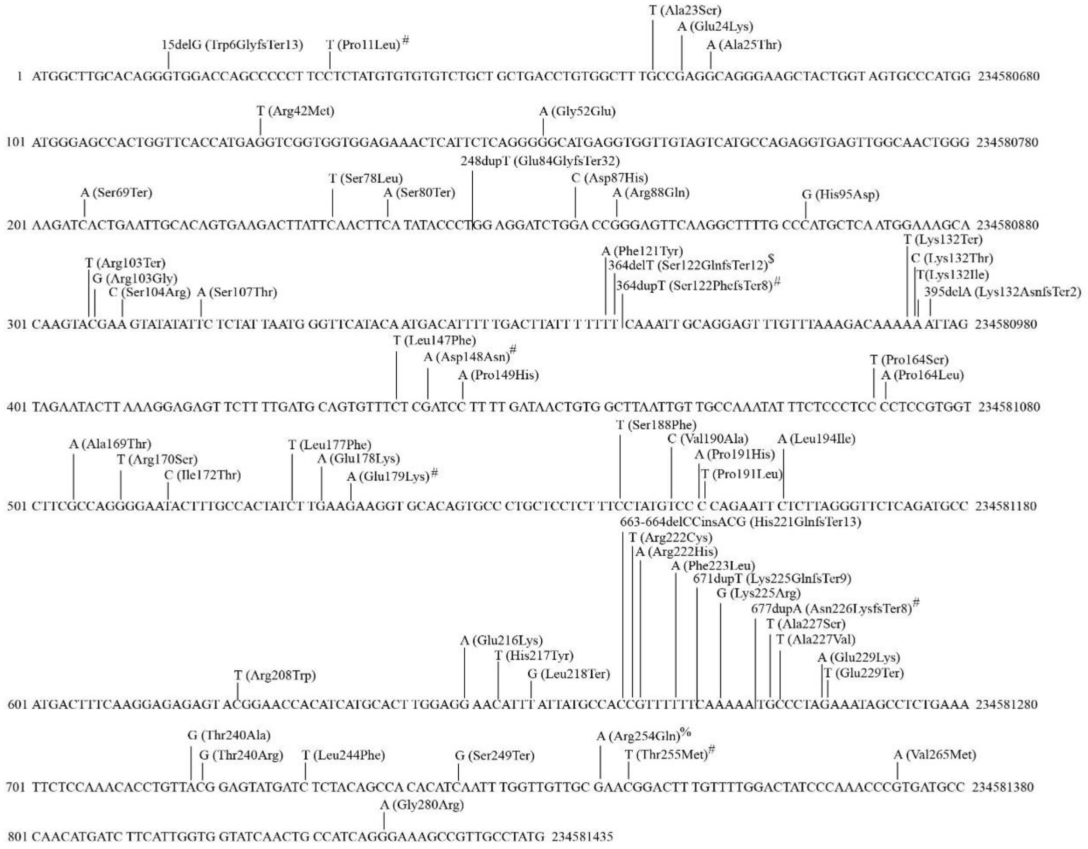

Figure S8. Mutations within the *UGT1A9* exon 1 in TCGA tumors. Data shown is the coding region of the *UGT1A9* exon 1 (855 bp) with genomic (GRCh37/hg19, right) and cDNA (NM_021027.3, left) positions. Mutations (missense, nonsense and small indels) and the resulting changes at the protein level (NP_066307.1) are indicated above the reference mRNA sequence. Recurrent mutations are indicated by # (twice), $ (three times), % (four times).

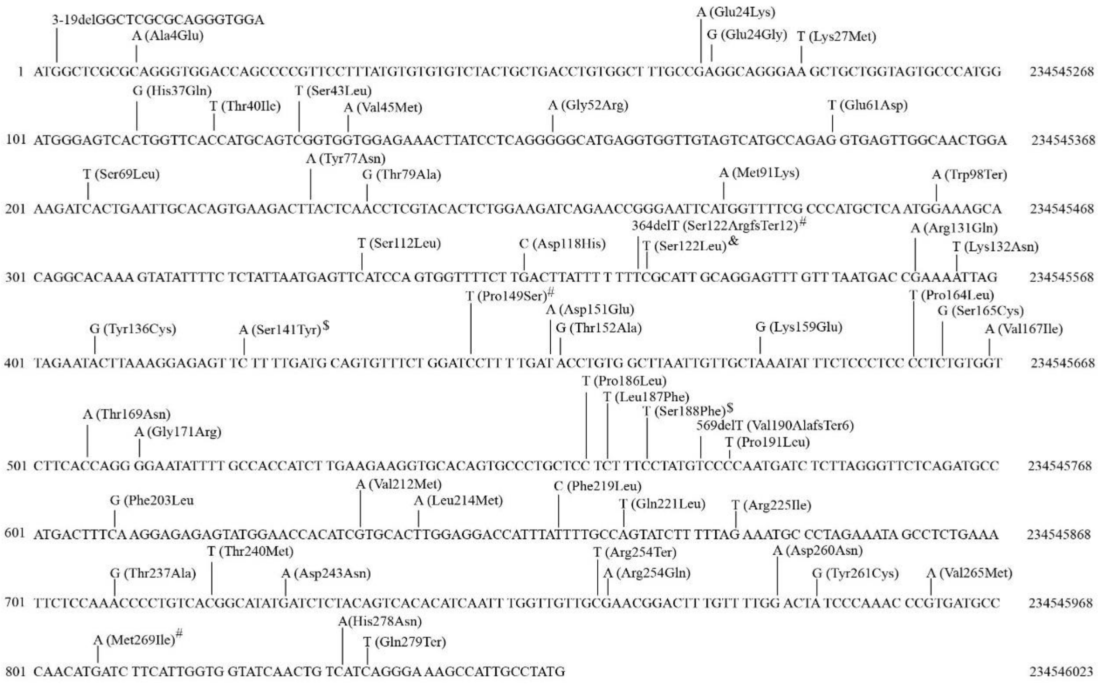

Figure S9. Mutations within the *UGT1A10* exon 1 in TCGA tumors. Data shown is the coding region of the *UGT1A10* exon 1 (855 bp) with genomic (GRCh37/hg19, right) and cDNA (NM_019075.4, left) positions. Mutations (missense, nonsense and small indels) and the resulting changes at the protein level (NP_061948.1) are indicated above the reference mRNA sequence. Recurrent mutations are indicated by # (twice), % (four times), & (five times)

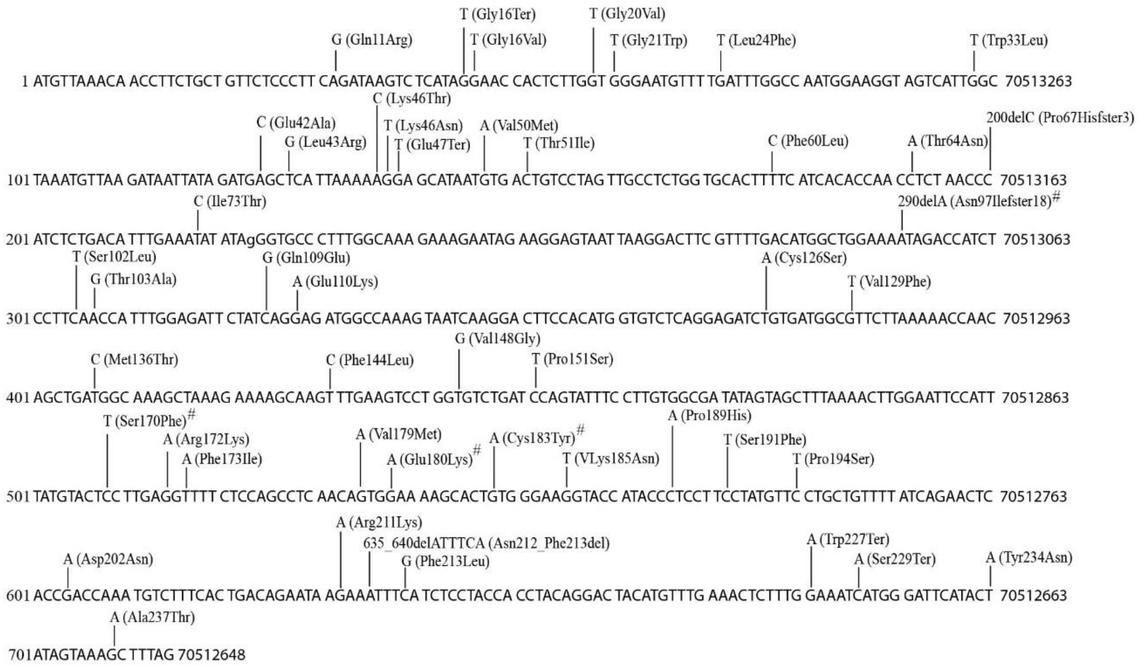

Figure S10. Mutations within the *UGT2A1* exon 1 in TCGA tumors. Data shown is the coding region of the *UGT2A1* exon 1 (715 bp) with genomic (GRCh37/hg19, right) and cDNA (NM_006798.5, left) positions. Mutations (missense, nonsense and small indels) and the resulting changes at the protein level (NP_006789.3) are indicated above the reference mRNA sequence. Recurrent mutations are indicated by # (twice).

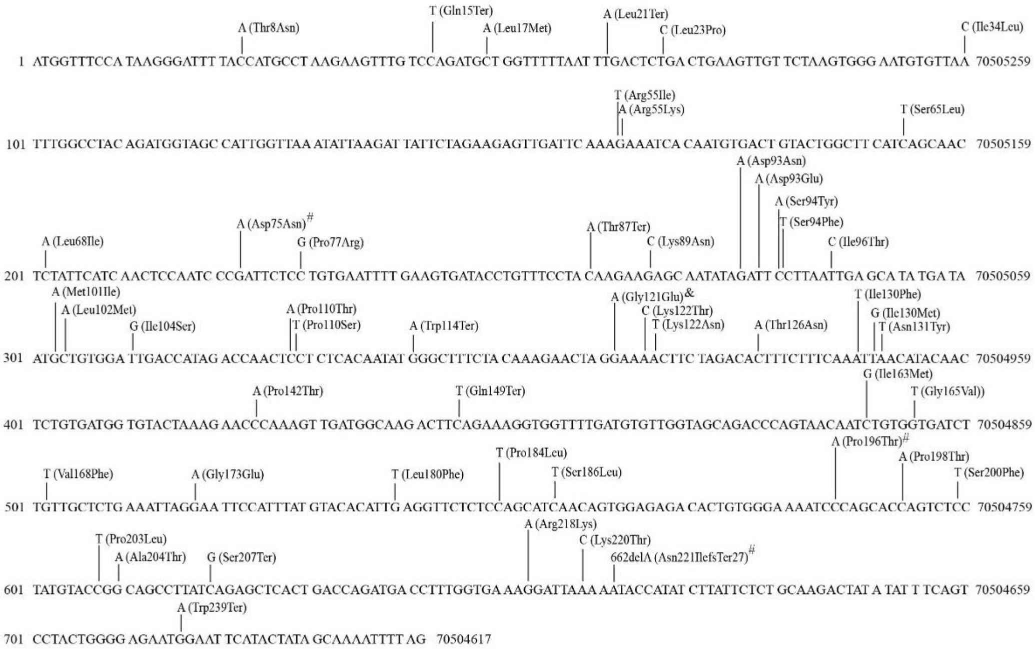

Figure S11. Mutations within the *UGT2A2* exon 1 in TCGA tumors. Data shown is the coding region of the *UGT2A2* exon 1 (742 bp) with genomic (GRCh37/hg19, right) and cDNA (NM_001105677.2, left) positions. Mutations (missense, nonsense and small indels) and the resulting changes at the protein level (NP_001099147.2) are indicated above the reference mRNA sequence.

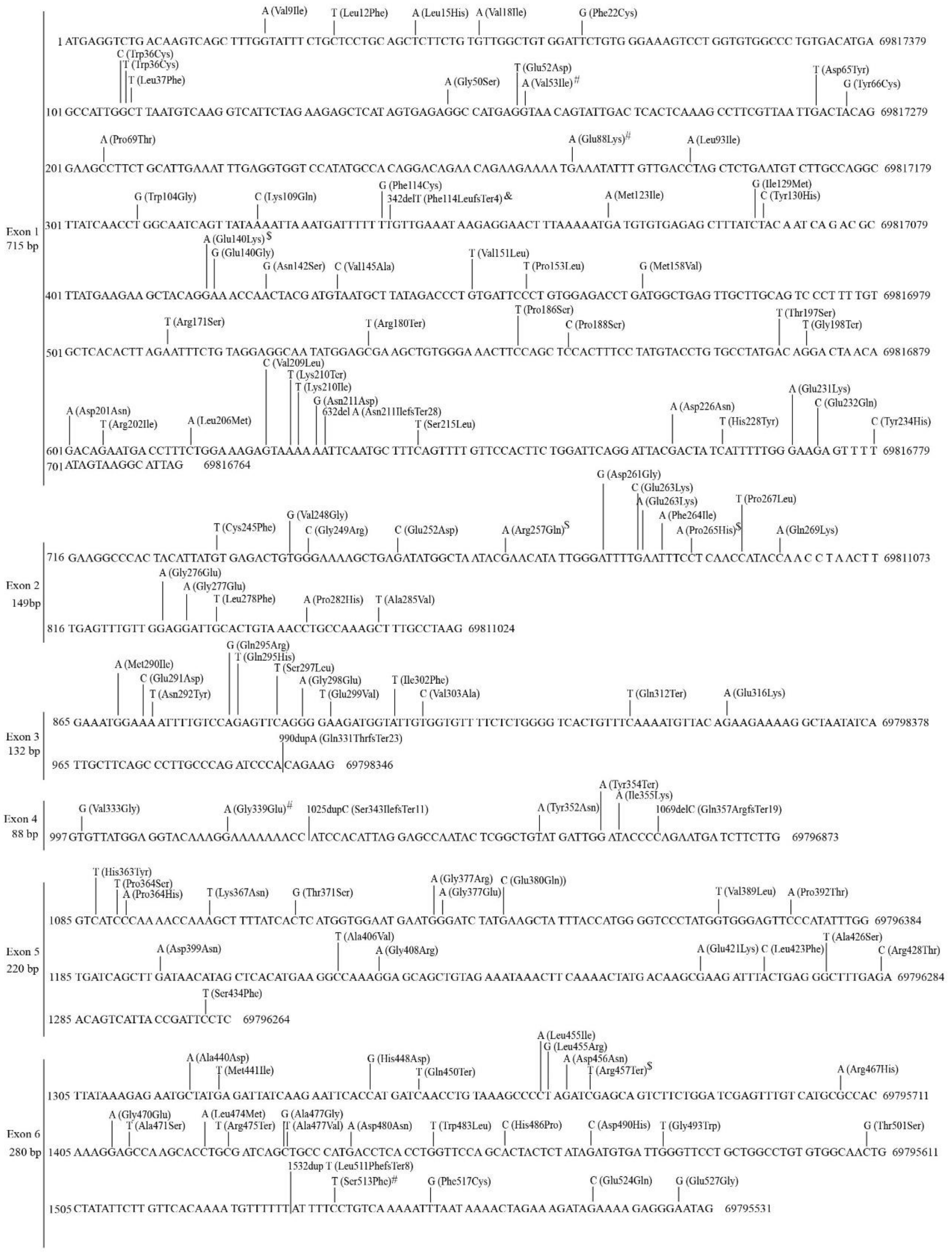

Figure S12. Mutations at the *UGT2A3* gene in TCGA tumors. Data shown is the coding region of the *UGT2A3* gene (6 exons, 1584 bp) with genomic (GRCh37/hg19, right) and cDNA (NM_024743.4, left) positions. Mutations (missense, nonsense and small indels) and the resulting changes at the protein level (NP_079019.3) are indicated above the reference mRNA sequence. Recurrent mutations are indicated by # (twice), $ (three times), & (five times).

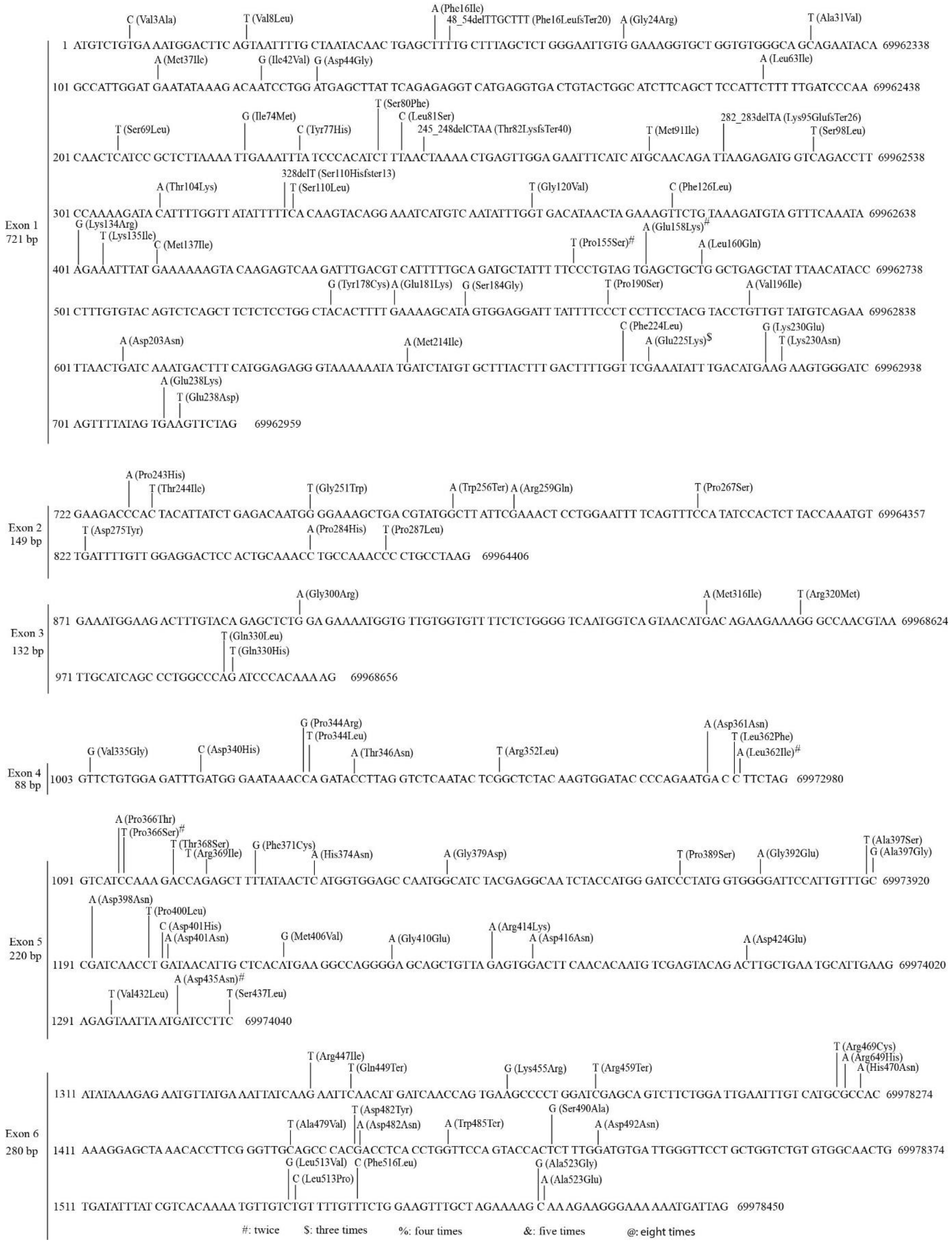

Figure S13. Mutations at the *UGT2B7* gene in TCGA tumors. Data shown is the coding region of the *UGT2B7* gene (6 exons, 1590 bp) with genomic (GRCh37/hg19, right) and cDNA (NM_001074.4, left) positions. Mutations (missense, nonsense and small indels) and the resulting changes at the protein level (NP_001065.2) are indicated above the reference mRNA sequence. Recurrent mutations are indicated by # (twice), $ (three times).

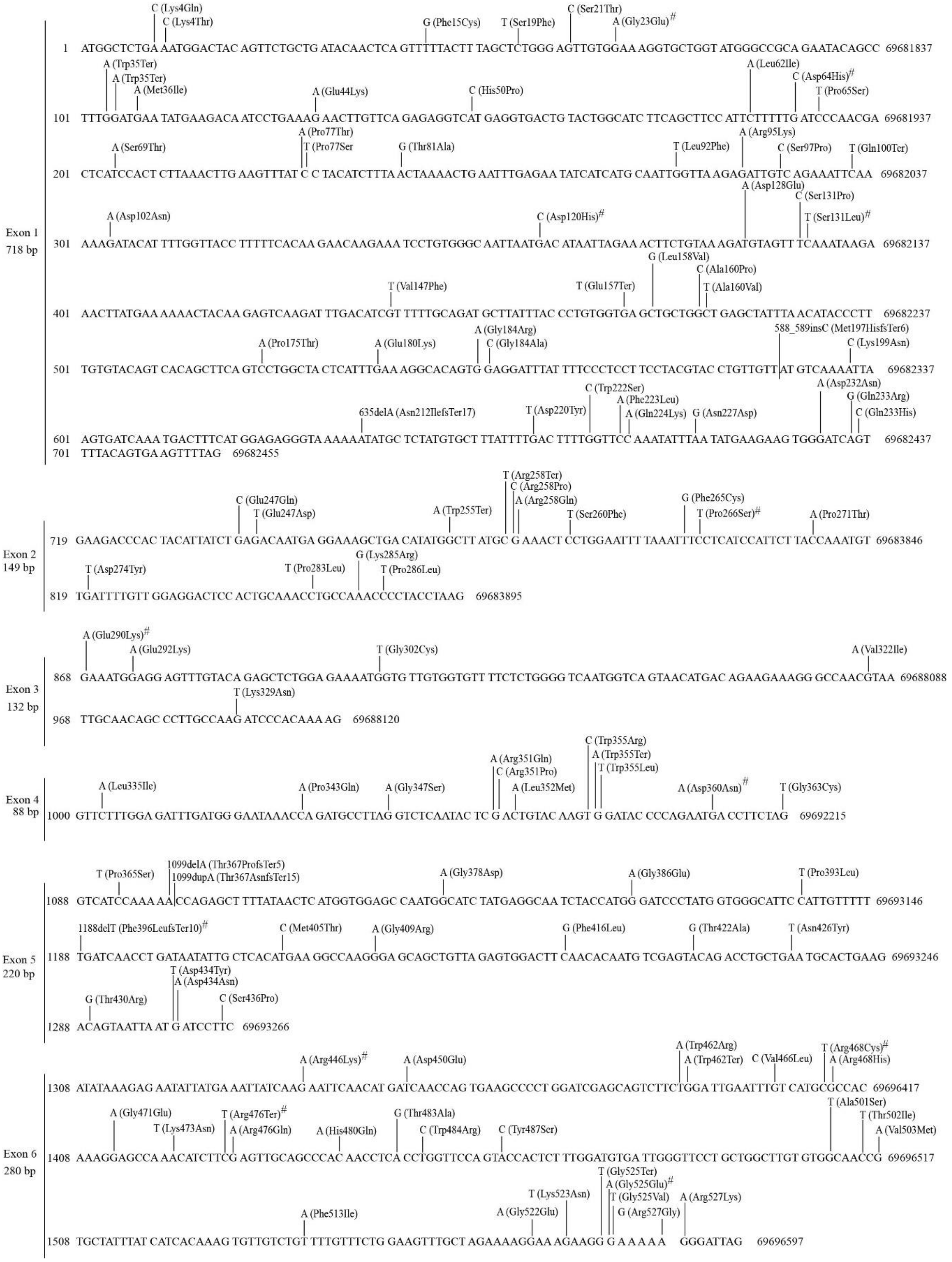

Figure S14. Mutations at the *UGT2B10* gene in TCGA tumors. Data shown is the coding region of the *UGT2B10* gene (6 exons, 1587 bp) with genomic (GRCh37/hg19, right) and cDNA (NM_001075.6, left) positions. Mutations (missense, nonsense and small indels) and the resulting changes at the protein level (NP_001066.1) are indicated above the reference mRNA sequence. Recurrent mutations are indicated by # (twice).

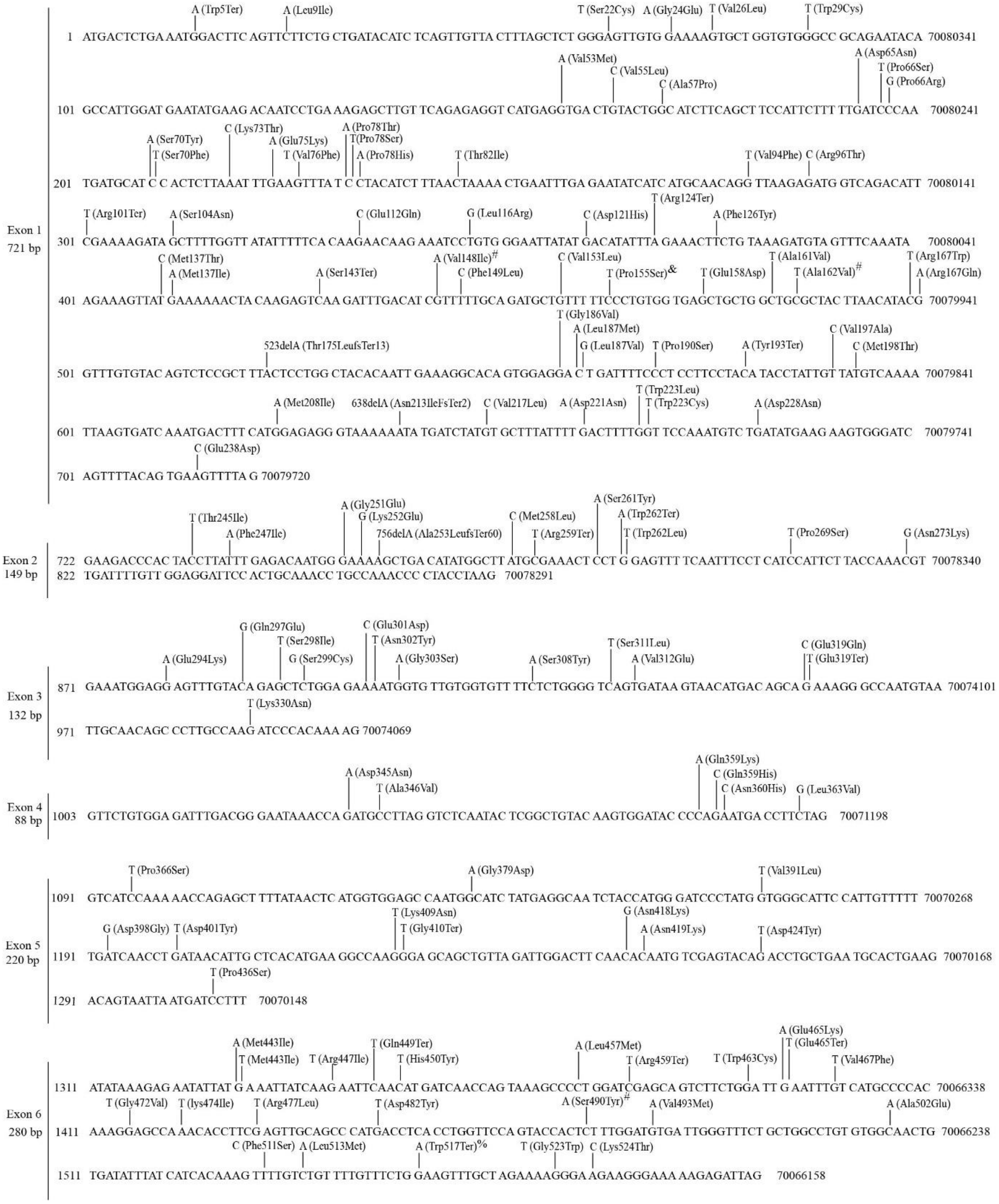

Figure S15. Mutations at the *UGT2B11* gene in TCGA tumors. Data shown is the coding region of the *UGT2B11* gene (6 exons, 1590 bp) with genomic (GRCh37/hg19, right) and cDNA (NM_001073.3, left) positions. Mutations (missense, nonsense and small indels) and the resulting changes at the protein level (NP_001064.1) are indicated above the reference mRNA sequence. Recurrent mutations are indicated by # (twice), & (five times).

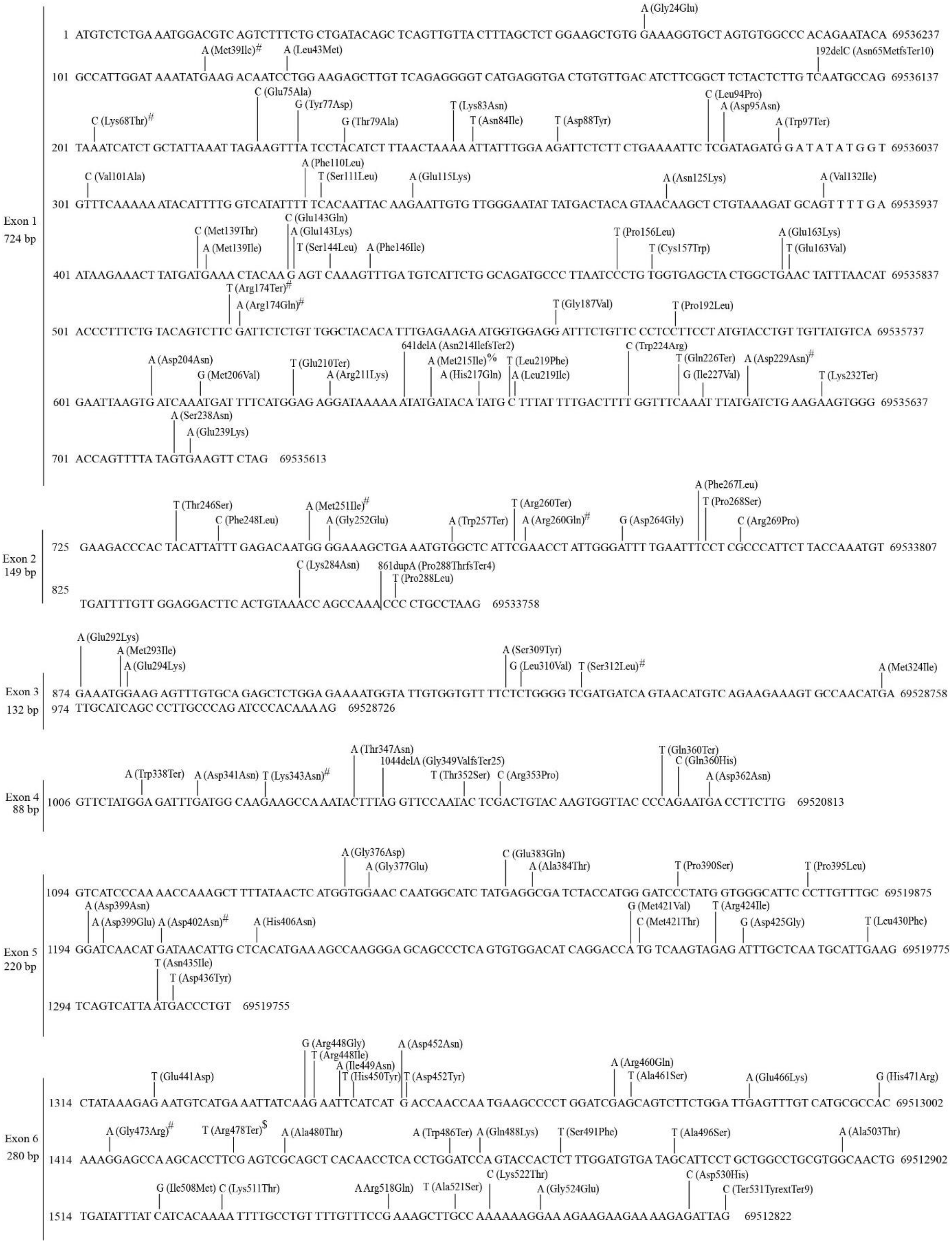

Figure S16. Mutations at the *UGT2B15* gene in TCGA tumors. Data shown is the coding region of the *UGT2B15* gene (6 exons, 1593 bp) with genomic (GRCh37/hg19, right) and cDNA (NM_001076.4, left) positions. Mutations (missense, nonsense and small indels) and the resulting changes at the protein level (NP_001067.2) are indicated above the reference mRNA sequence. Recurrent mutations are indicated by # (twice), $ (three times), % (four times).

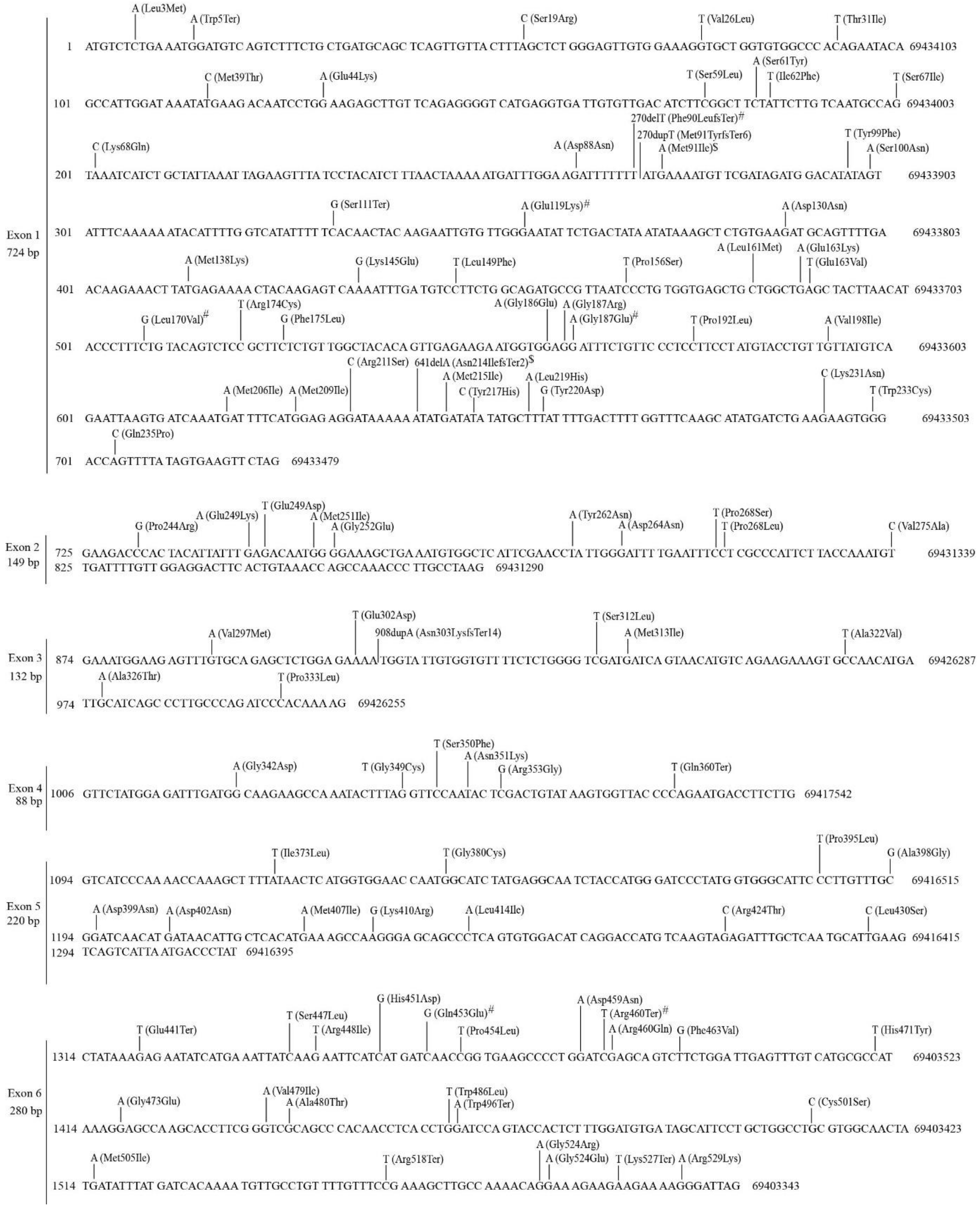

Figure S17. Mutations at the *UGT2B17* gene in TCGA tumors. Data shown is the coding region of the *UGT2B17* gene (6 exons, 1593 bp) with genomic (GRCh37/hg19, right) and cDNA (NM_001077.4, left) positions. Mutations (missense, nonsense and small indels) and the resulting changes at the protein level (NP_001068.1) are indicated above the reference mRNA sequence. Recurrent mutations are indicated by # (twice), $ (three times).

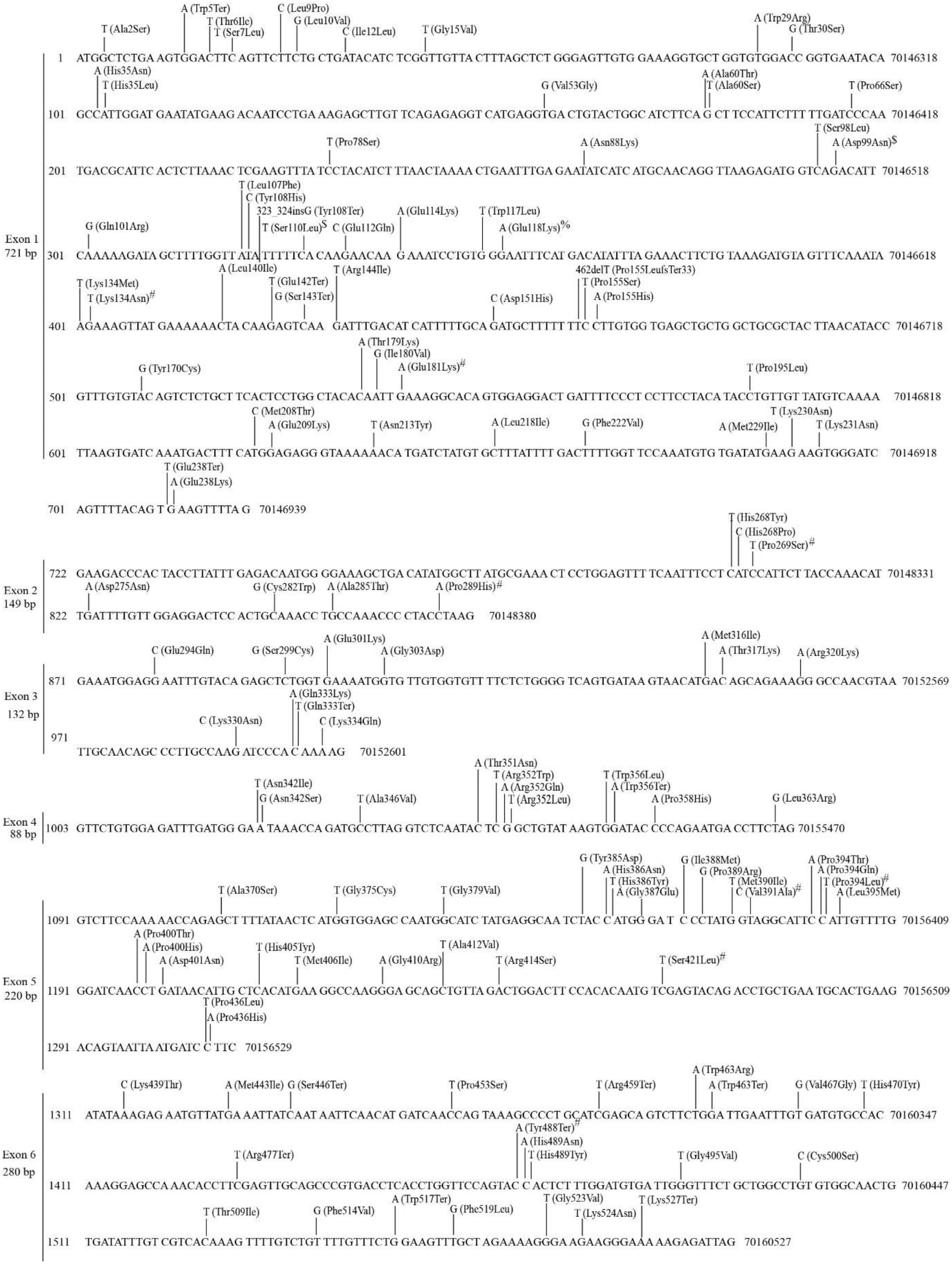

Figure S18. Mutations at the *UGT2B28* gene in TCGA tumors. Data shown is the coding region of the *UGT2B28* gene (6 exons, 1590 bp) with genomic (GRCh37/hg19, right) and cDNA (NM_053039.2, left) positions. Mutations (missense, nonsense and small indels) and the resulting changes at the protein level (NP_444267.1) are indicated above the reference mRNA sequence. Recurrent mutations are indicated by # (twice), $ (three times), % (four times).

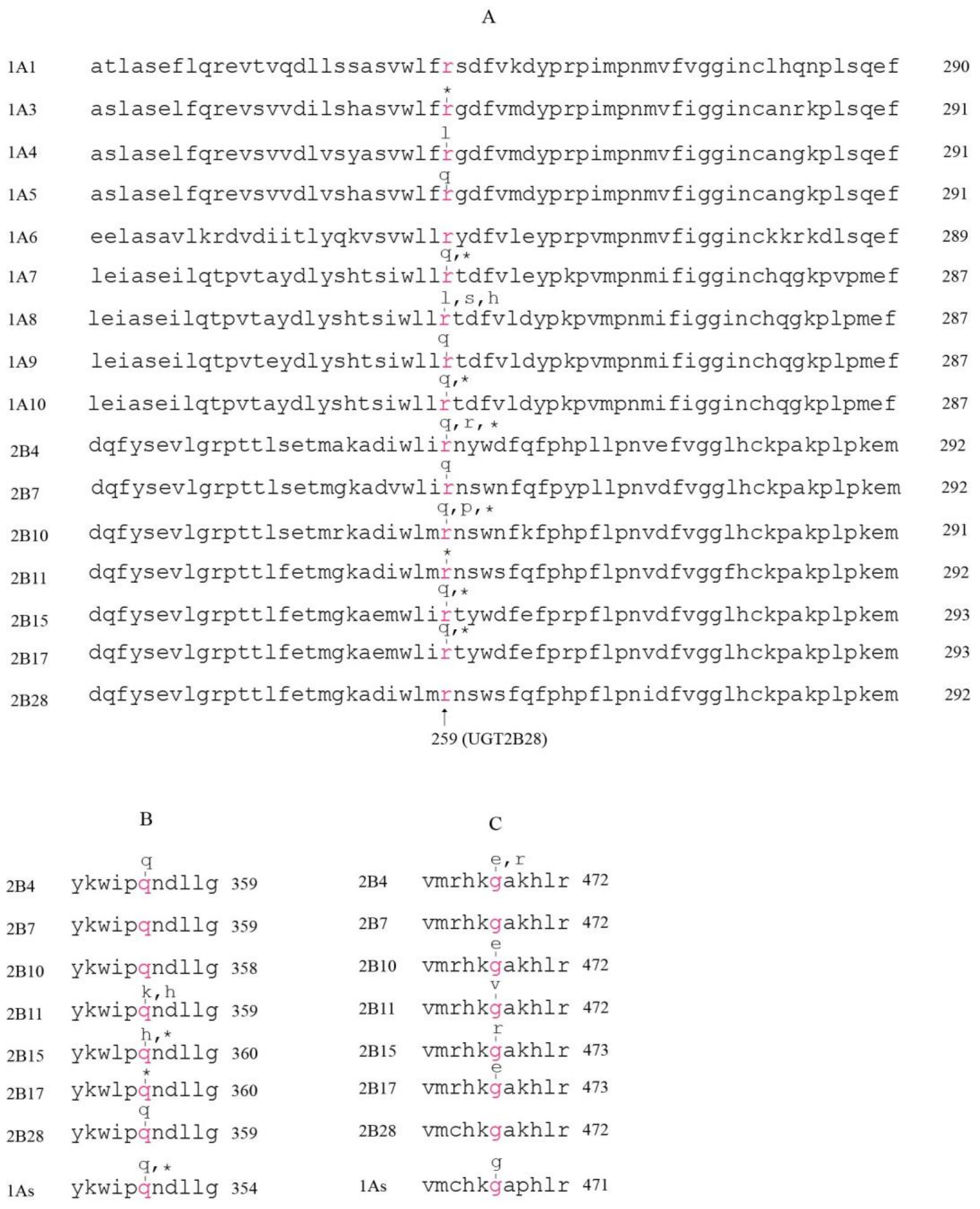

Figure S19. Mutations within the codons of conserved amino acids across the UGT1A and UGT2B family enzymes. The amnio acid sequences of the UGT1A and UGT2B enzymes are aligned using the Clustal Omega program. Data shown are the sequence alignments surrounding the conserved amnio acids [in Red, positions given at the bottom (A) or at the left (B, C)] whose codons were mutated in TCGA tumors. As indicated above the conserved amnio acids, these mutations lead to 1) amnio acid substitution (missense), 2) no change in amnio acid sequence (silent mutation) or 3) premature stop codons (nonstop mutation, specified by *).

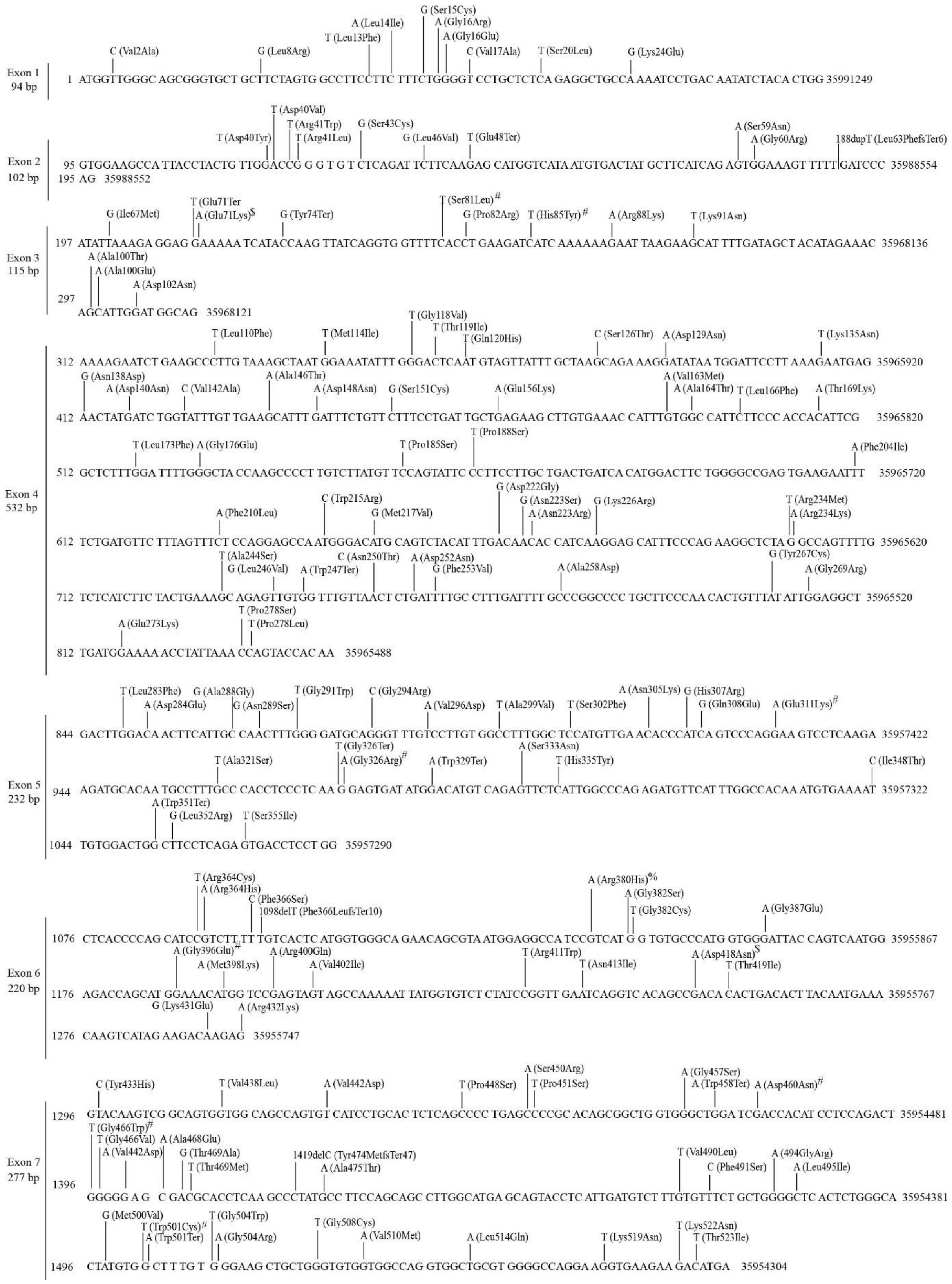

Figure S20. Mutations at the *UGT3A1* gene in TCGA tumors. Data shown is the coding region of the *UGT3A1* gene (7 exons, 1572 bp) with genomic (GRCh37/hg19, right) and cDNA (NM_152404.4, left) positions. Mutations (missense, nonsense and small indels) and the resulting changes at the protein level (NP_689617.3) are indicated above the reference mRNA sequence. Recurrent mutations are indicated by # (twice), $ (three times), % (four times), & (five times).

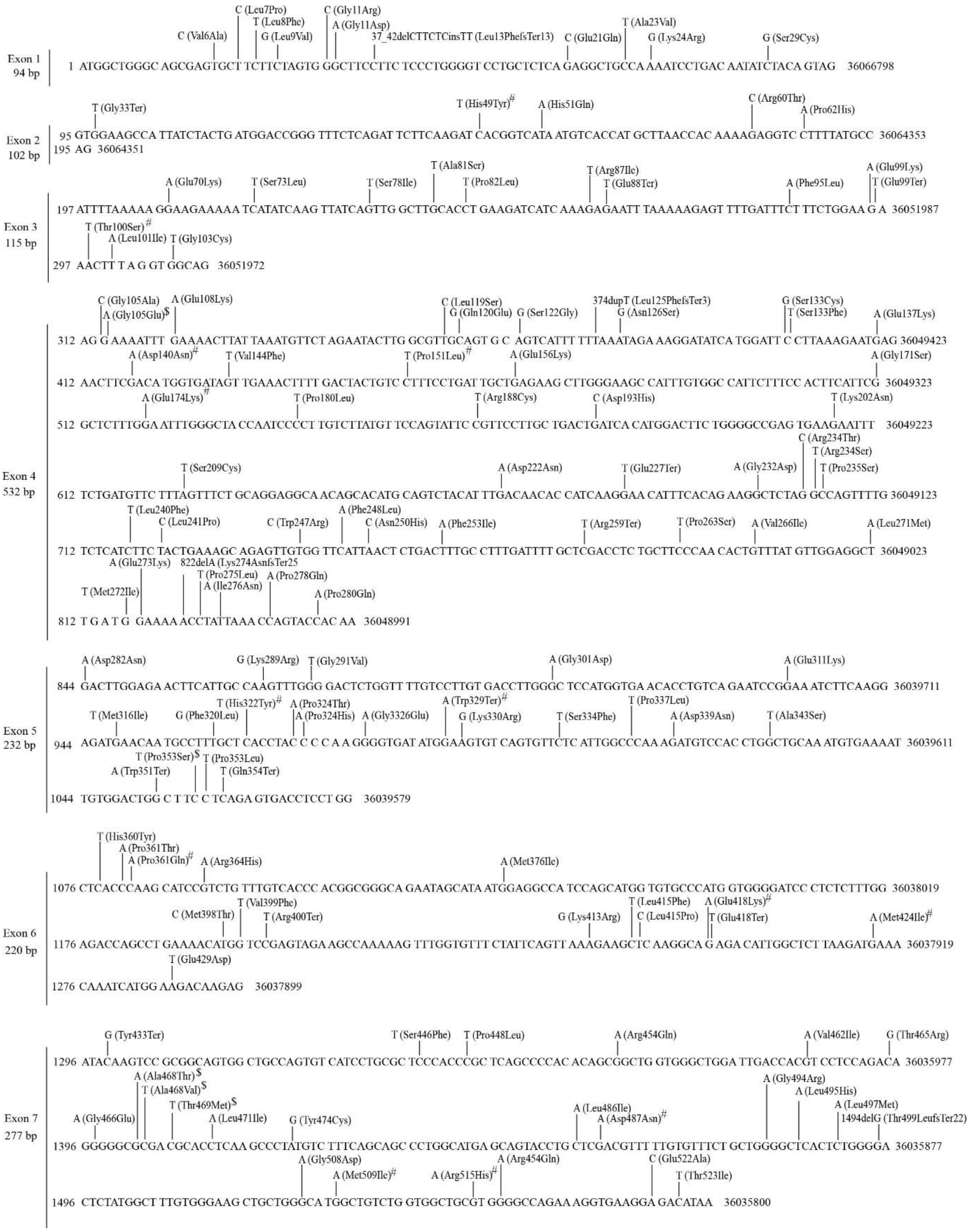

Figure S21. Mutations at the *UGT3A2* gene in TCGA tumors. Data shown is the coding region of the *UGT3A2* gene (7 exons, 1590 bp) with genomic (GRCh37/hg19, right) and cDNA (NM_174914.4, left) positions. Mutations (missense, nonsense and small indels) and the resulting changes at the protein level (NP_777574.2) are indicated above the reference mRNA sequence. Recurrent mutations are indicated by # (twice), $ (three times), % (four times).

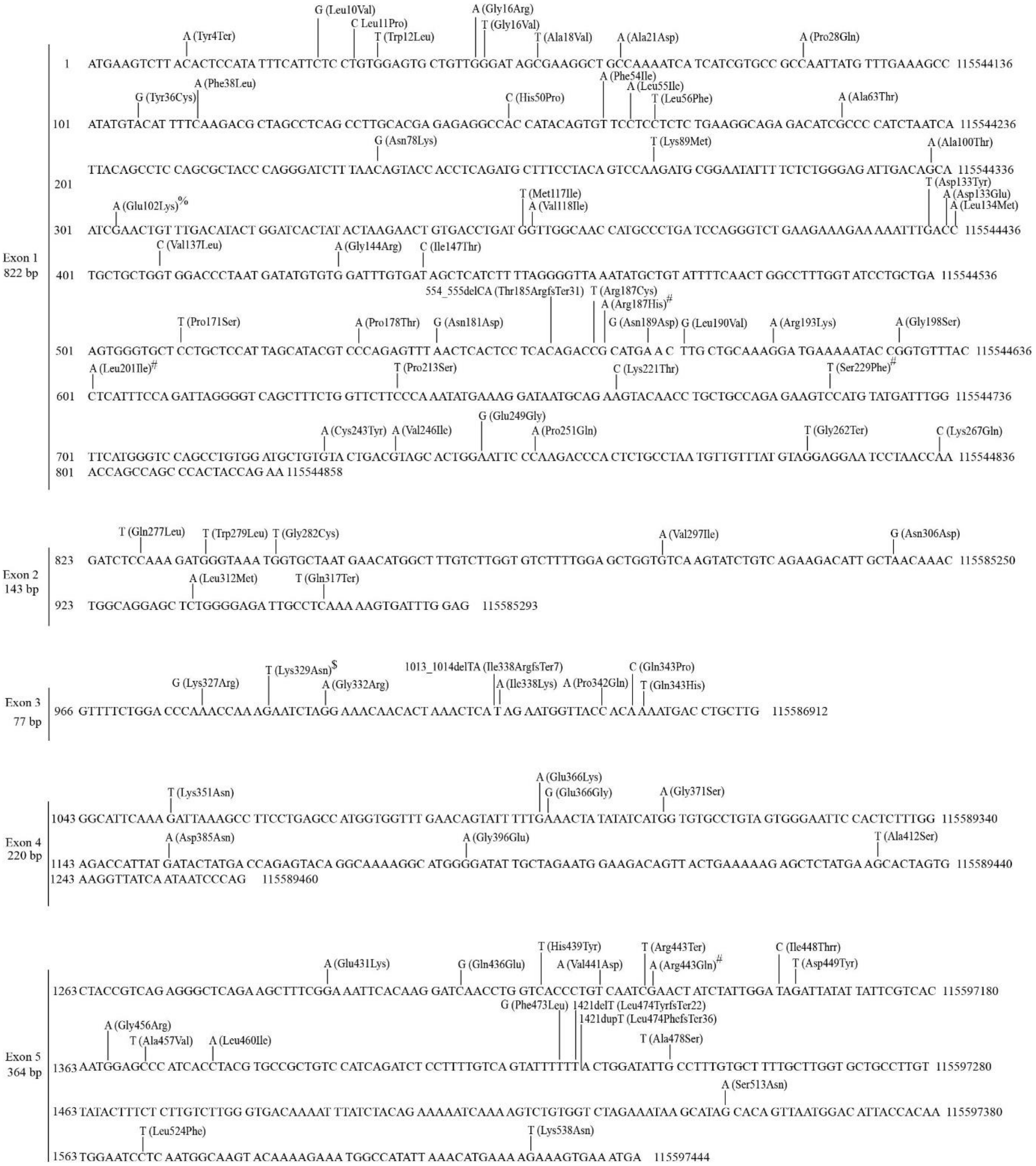

Figure S22. Mutations at the *UGT8* gene in TCGA tumors. Data shown is the coding region of the *UGT8* gene (5 exons, 1590 bp) with genomic (GRCh37/hg19, right) and cDNA (NM_001128174.3, left) positions. Mutations (missense, nonsense and small indels) and the resulting changes at the protein level (NP_001121646.2) are indicated above the reference mRNA sequence. Recurrent mutations are indicated by # (twice), $ (three times), % (four times).

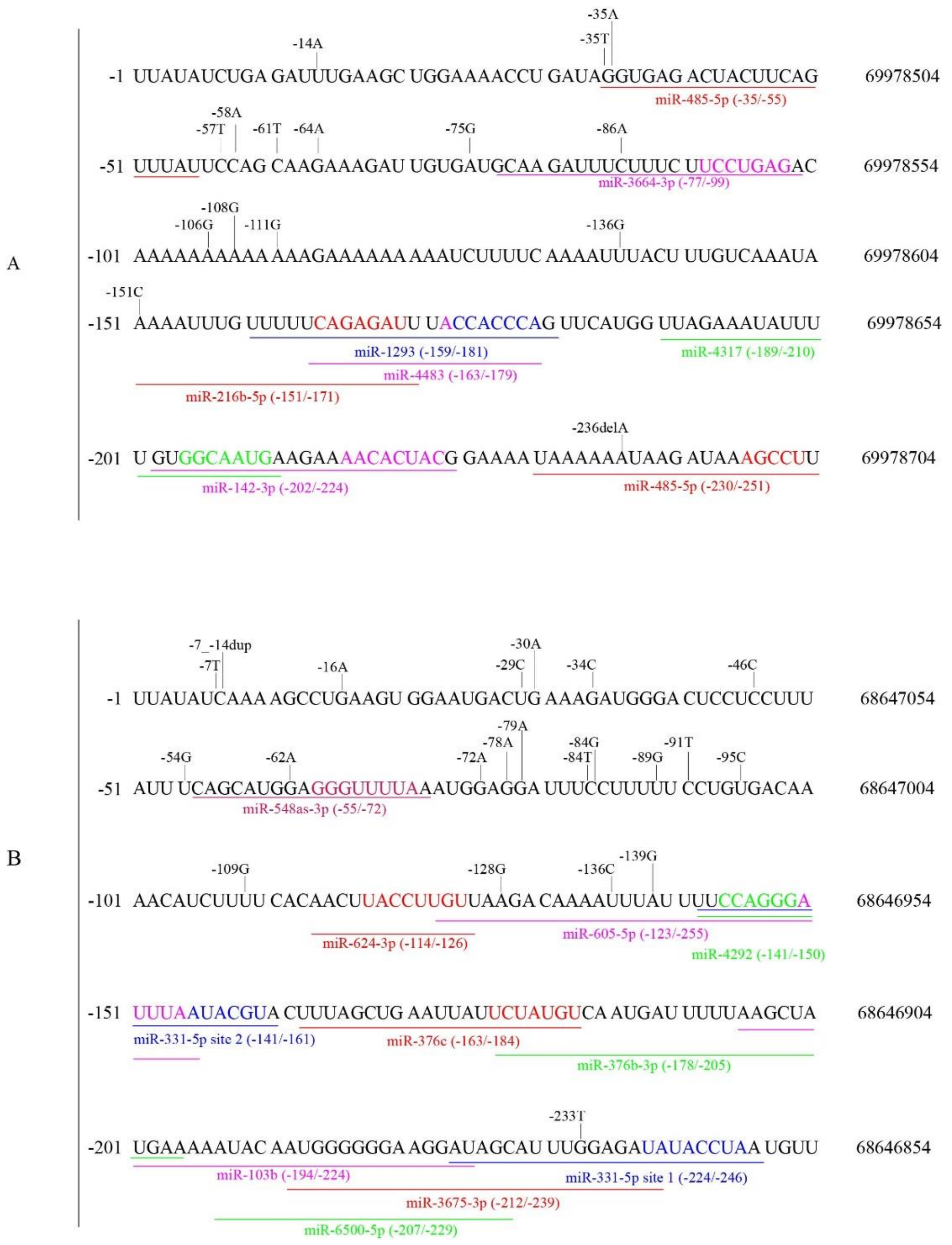

Figure S23. Somatic mutations within/adjacent to known miRNA target sites at UGT 3′UTRs in TCGA tumors. Data shown are the first 250-nt sequence of the 3′ UTR region for UGT2B7 (A) or UGT2B15 (B) with positions indicated at the cDNA (left) and genomic (GRCh37/hg19) (right) levels. Somatic mutations identified in TCGA tumors are given above the sequences. The sequences underlined and colored are known functional miRNA target sites (positions indicated in the BRACKETs) with the miRNA seed target sites highlighted in matched colors.
