# Supplementary material for: The Somatic Mutation Landscape of UDP-Glycosyltransferase (UGT) Genes in Human Cancers
Source: Cancers (Basel). 2022 Nov 21;14(22):5708. doi: 10.3390/cancers14225708 (PMC9688768; doi:10.3390/cancers14225708)
Supplement: Supplementary file 1 [file cancers-14-05708-s001.zip › Table S10.pdf]

Table S10. Mutations of *UGT* genes found in both TCGA tumors and CCLE cell lines

UGT genes	Mutations	TCGA tumors	CCLE
1A1	c.676G>A (Val226Ile)	SKCM-Metastatic	Colorectal cancer (LS180)
1A4	c.517dupT (Trp173LeufsTer7)	STAD, UCEC	Colorectal cancer (CW2, SW48)
1A5	c.850G>A (Gly284Arg)	UCEC	Endometrial cancer (HEC151)
	c.725_726dupAG (Val243ArgfsTer21)	ARC	Endometrial cancer (HEC265)
1A6	c.29G>T (Arg10Ile)	HNSC	Colorectal cancer (SNU81)
	c.616del (Ser206ProfsTer38)	STAD	Mixed cancer types (RHJT)
	c.831C>G (Ile277Met)	STAD	Glioma (LN428)
1A7	c.8G>A (Arg3His)	UCS	Colorectal cancer (SNU407)
	c.364delT (Ser122GlnfsTer12)	COAD, STAD	Colorectal cancer (KM12); Hodgkin Lymphoma (SUPHD1)
	c.761G>A (Arg254Gln)	COAD, SKCM primary	Cancer of unknown primary (HCC2998)
	c.760C>T (Arg254Ter)	OV	Small cell lung cancer (NCIH446)
	c.577C>A (Leu193Ile)	LUAD	Renal cell carcinoma (SNU1272); Mature B-cell neoplasms (KMS26); Esophagogastric cancer (SNU1)
	c.719C>T (Thr240Met)	ESCA, LUSC, PRAD	Non-small cell lung cancer (NCIH1355); Cancer of unknown primary (NCIH1688)
1A8	c.200G>A (Gly67Glu)	SKCM-metastatic	Non-small cell lung cancer (NCIH2405)
	c.308G>A (Arg103Gln)	HNSC	T-Lymphoblastic Leukemia/Lymphoma (RPMI8402)
	c.391C>T (Arg131Ter)	LUSC, STAD	Endometrial cancer (HEC1A); Cancer of unknown primary (HEC1)
	c677delA (Asn226MetfsTer3)	UCEC	Endometrial cancer (SNGM)
1A9	c.364delT (Ser122GlnfsTer12)	UCEC	T-Lymphoblastic Leukemia/Lymphoma (MOLT4, JURKAT)
	c.442G>A (Asp148Asn)	COAD, SKCM-metastatic	Endometrial cancer (JHUEM1)
	c.685G>A (Glu229Lys)	GBM	Melanoma (WM983B)
1A10	c.511G>A (Gly171Arg)	SKCM-metastatic	Melanoma (CHL1)
1A E2-5	c.1566delA (Arg524GlufsTer22)	BRCA, COAD, STAD	Endometrial cancer (RL952, HEC59); T-Lymphoblastic Leukemia/Lymphoma (MOLT4, MOLT3)
			Colorectal cancer (SNU1040)
2A2	c.362G>A (Gly121Glu)	LIHC, SKCM (Primary, Metastatic)	Melanoma (CP66MEL)
	c.557C>T (Ser186Leu)	SKCM-metastatic	Bladder cancer (5637)
2A E2-6	c.845C>A (Pro282His)	KIRP	Non-small cell lung cancer (HCC827)
2A3	c.342delT (Phe114LeufsTer4)	CESC, COAD, STAD	Colorectal cancer (CW2, SNU407); Cancer of unknown primary (DG75)
	c.890C>T (Ser297Leu)	SKCM-metastatic	Cancer of unknown primary (TGW)
	c.1195G>A (Asp399Asn)	SKCM-metastatic	Mature B-cell neoplasms (HUNS1)
	c.1448G>T (Trp483Leu)	KIRC	Cancer of unknown primary (LAN6)
	c.1532dupT (Leu511PhefsTer8)	THYM	Endometrial cancer (EN)
2B4	c.133G>A (Glu45Lys)	LUAD, SKCM (Primary, Metastatic)	Melanoma (IPC298)
	c.340G>T (Glu114Ter)	UCEC	Endometrial cancer (JHUEM7)
	c.541G>A (Glu181Lys)	SKCM-metastatic	Melanoma (IGR39, IGR37)
	c.638delA (Asn213IlefsTer2)	STAD	Colorectal cancer (CW2)
	c.1160G>A (Gly387Glu)	SKCM-metastatic	Cancer of unknown primary (MCC13)
	c.1306C>T (Pro436Ser)	SKCM-metastatic	Melanoma (COLO792)
	c.1340G>T (Arg447Ile)	UCEC	Endometrial cancer (ESS1)

	c.1405C>T (Arg469Cys)	LGG	T-Lymphoblastic Leukemia/Lymphoma (MOLT16)
	c.1558A>G (Arg520Gly)	LUSC	Blood cancer (KCL22)
2B7	c.1096C>A (Pro366Thr)	PRAD	Cancer of unknown primary (BB49HNC
	c.1192G>A (Asp398Asn)	LGG	Colorectal cancer (RCM1); Blood cancer (KCL22)
	c.1340G>T (Arg447Ile)	UCEC	Cancer of unknown primary (JEG3)
	c.1375C>T (Arg459Ter)	KICH	Colorectal cancer (HCT116)
	c.1444G>A (Asp482Asn)	SKCM-primary	Endometrial cancer (JHUEM7)
2B10	c.104G>A (Trp35Ter)	SKCM-metastatic	Cancer of unknown primary (HCC515)
	c.184C>A (Leu62Ile)	OV	Melanoma (COLO679, C32); Small cell lung cancer (NCIH2081)
	c.635delA (Asn212IlefsTer17)	STAD	Colorectal cancer (LS411N, SW48)
	c.1003C>A (Leu335Ile)	BRCA	Endometrial cancer (ESS1)
	c.1300G>A (Asp434Asn)	SKCM-metastatic	Cancer of unknown primary (NCIH1688)
	c.1403G>A (Arg468His)	HNSC	Cancer of unknown primary (HELA)
	c.1412G>A (Gly471Glu)	SKCM-metastatic	Mixed cancer types (MM370)
	c.1565G>A (Gly522Glu)	SKCM-metastatic	Cancer of unknown primary (M14)
2B11	c.71G>A (Gly24Glu)	SKCM-primary	Cancer of unknown primary (MCC13)
	c.500G>A (Arg167Gln)	LUAD	Cancer of unknown primary (EB3)
	c.923C>A (Ser308Tyr)	UCEC	Cancer of unknown primary (HCC2998)
	c.932C>T (Ser311Leu)	HNSC	Prostate cancer (LNCAPCLONEFGC)
	c.1270G>T (Asp424Tyr)	LUAD	Non-small cell lung cancer (EKVX)
	c.1306C>T (Pro436Ser)	SKCM-metastatic	Mixed cancer types (MM386)
	c.1567G>T (Gly523Trp)	COAD	Pancreatic cancer (HS766T)
2B15	c.117G>A (Met39Ile)	LUAD, SKCM-metastatic	Cancer of unknown primary (TASK1)
	c.283G>A (Asp95Asn)	SKCM-metastatic	Melanoma (HS939T)
	c.332C>T (Ser111Leu)	SKCM-metastatic	Melanoma (MEWO)
	c.521G>A (Arg174Gln)	CESC, COAD	Colorectal cancer (SNU81)
	c.1323G>T (Glu441Asp)	STAD	Mature B-cell neoplasms (NAMALWA)
	c.1354G>T (Asp452Tyr)	LUAD	Non-small cell lung cancer (HCC1438)
	c.1458G>A (Trp486Ter)	SKCM-metastatic	Melanoma (MEWO)
2B17	c.790G>A (Asp264Asn)	SKCM-primary	Cancer of unknown primary (MDAMB330)
2B28	c.329C>T (Ser110Leu)	SKCM-metastatic	Cancer of unknown primary (SW684)
	c.352G>A (Glu118Lys)	SKCM (primary, Metastatic)	Cancer of unknown primary (SW684)
	c.462delT (Pro155Leufster33)	STAD	Colorectal cancer (RKO)
	c.463C>T (Pro155Ser)	SKCM-metastatic	Cancer of unknown primary (NCIH1770)
	c.901G>A (Glu301Lys)	SKCM-metastatic	Mixed cancer types (MM370)
	c.1198C>A (Pro400Thr)	LUAD	Non-small cell lung cancer (NCIH1623)
	c.1262C>T (Ser421Leu)	GBM, LUSC	Esophagogastric Cancer (KYSE140)
	c.1329G>A (Met443Ile)	SKCM-metastatic	Esophagogastric Cancer (SNU1); Melanoma (MEWO)
	c.1375C>T (Arg459Ter)	SKCM-metastatic	Cancer of unknown primary (SW684)
3A1	c.37C>T (Leu13Phe)	SKCM-metastatic	Colorectal cancer (SNU175); Cancer of unknown primary (SW872)
	c.70A>G (Lys24Glu)	LUAD	Cancer of unknown primary (LNZTA3WT4)

	c.211G>A (Glu71Lys)	SKCM (primary, Metastatic)	Colorectal cancer (MDST8)
	c.490G>A (Ala164Thr)	COAD	Mature B-cell neoplasms (U266B1)
	c.1378G>A (Asp460Asn)	BLCA, SKCM-metastatic	Colorectal cancer (SNU81)
3A2	c.218C>T (Ser73Leu)	SKCM-metastatic	Cancer of unknown primary (SARC9371)
	c.262G>T (Glu88Ter)	UCEC	Colorectal cancer (HT115)
	c.364A>G (Ser122Gly)	LUSC	Cancer of unknown primary (NCIH513)
	c.452C>T (Pro151Leu)	LUSC, SKCM-metastatic	Melanoma (WM793)
	c.1091G>A (Arg364His)	GBM	Endometrial cancer (HEC1A)
	c.1406C>T (Thr469Met)	BRCA, GBM, LGG, LUAD	Cancer of unknown primary (BN6)
	c.1544G>A (Arg515His)	COAD, GBM	Colorectal cancer (SW403)
UGT8	c.187G>A (Ala63Thr)	STAD	B-Lymphoblastic Leukemia/Lymphoma (NAML6)
	c.559C>T (Arg187Cys)	LUSC	Cancer of unknown primary (D247MG)
	c.1053G>T (Lys351Asn)	UCEC	Endometrial cancer (RL952)
	c.1421dupT (Leu474PhefsTer36)	COAD	Esophagogastric Cancer (2313287)
