# Supplementary material for: The Somatic Mutation Landscape of UDP-Glycosyltransferase (UGT) Genes in Human Cancers
Source: Cancers (Basel). 2022 Nov 21;14(22):5708. doi: 10.3390/cancers14225708 (PMC9688768; doi:10.3390/cancers14225708)
Supplement: Supplementary file 1 [file cancers-14-05708-s001.zip › Table S6.pdf]

Table S6. Mutations at 5’ untranslated regions (UTRs) of *UGT* genes in TCGA tumors

1A8	1A10	1A9	1A4	1A6	1A3	1A1	2A1	2A3	2B4	2B7	2B10	2B11	2B15	2B17	2B28	3A1	3A2
c-58G>A<br>c-17C>T	c.-17C>T<br>c.-11G>A<br>c.-1C>T	c.-39T>C<br>c.-32C>T	c.-25T>C	c.-117G>C<br>c.-101C>A<br>c.-96G>A<br>c.89A>G<br>c.-74G>A<br>c.-53G>A<br>c.-38_-37delAA<br>c.-36C>T<br>c.-34C>T<br>c.-25C>T<br>c.-4C>T	c.-3G>A	c.-10C>A<br>c.-3G>A	c.-39G>T<br>c.-25C>A<br>c.-20C>A<br>c.-11C>G	c.-25A>T<br>c.-4C>T	c.-26G>T<br>c.-26G>A<br>c.-18G>T<br>c.-8G>A	c.-27G>A<br>c.-1G>A	c.-26G>A<br>c.-17T>A<br>c.-2G>A	c.-7C>T<br>c.-1G>C	c.-7G>A<br>c.-4C>A	c.-2G>A	c.-1G>A	c.-159>A<br>c.-112C>T<br>c.-90C>A<br>c.-73C>T<br>c.-72G>A<br>c.-70G>A<br>c.-52G>A<br>c.-51G>A<br>c.-49G>A<br>c.-46G>T<br>c.-43C>T<br>c.-37G>A<br>c.-28G>C<br>c.-22C>T<br>c.-22C>A<br>c.-14C>T<br>c.-4G>T	c.-81G>C<br>c.-78C>T<br>c.-76G>C<br>c.-62C>A<br>c.-14C>T<br>c.-13C>T<br>c.-12G>C<br>c.-11T>C<br>c.-2G>A

Table S6. Mutations at 5’ untranslated regions (UTRs) of *UGT* genes in TCGA tumors
