# Supplementary material for: The Somatic Mutation Landscape of UDP-Glycosyltransferase (UGT) Genes in Human Cancers
Source: Cancers (Basel). 2022 Nov 21;14(22):5708. doi: 10.3390/cancers14225708 (PMC9688768; doi:10.3390/cancers14225708)
Supplement: Supplementary file 1 [file cancers-14-05708-s001.zip › Table S7.pdf]

Table S7. Mutations at 3’ untranslated regions (UTRs) of *UGT* genes in TCGA tumors. Mutations in functional miRNA seed target sites are in bold.

1As	2As	2A3	2B4	2B7	2B10	2B11	2B15	2B28	3A1	3A2	UGT8
c.*1G>C	c.*15G>A	c.*5T>A	c.*4C>T	c.*14T>C	c.*4T>A	c.*5G>A	c.*7C>T	c.*8T>G	c.*7G>A	c.*6G>T	c.*13G>C
c.*37T>C	c.*15G>A	c.*26G>C	c.*5G>A	c.*35G>T	c.*8T>G	c.*23G>A	c.*7_*14dupCAAAAGCC	c.*23G>A	c.*7G>T	c.*20G>A	c.*20G>T
c.*46A>G	c.*20A>G	c.*58G>A	c.*16G>A	c.*35G>A	c.*9G>C	c.*64G>A	c.*16G>A	c.*31G>A	c.*17G>T	c.*20G>A	c.*38C>G
c.*65G>T	c.*21T>C	c.*63dupT	c.*23G>T	c.*57C>T	c.*11G>A	c.*73G>T	c.*16G>A	c.*35G>A	c.*18G>T	c.*21G>A	c.*75A>G
**c.*70A>T**	c.*27_*28insA	c.*67G>T	c.*24G>A	c.*58C>A	c.*26_*42del	c.*73G>T	c.*29T>C	c.*122C>G	c.*23G>A	c.*23dupG	c.*274G>C
**c.*74T>A**	c.*45T>C	c.*67G>A	c.*38G>T	c.*61C>T	c.*58C>A	c.*75T>C	c.*29T>C	c.*167C>G	c.*25G>T	c.*28T>C	c.*379G>T
c.*78A>C		c.*96T>C	c.*38G>T	c.*64G>A	c.*78C>A	c.*86G>T	c.*30G>A	c.*171C>T	c.*26G>T	c.*35G>T	
c.*83T>C		c.*97C>A	c.*41C>A	c.*75A>G	c.*84T>G	c.*99C>A	c.*34G>C	c.*194G>A	c.*33T>G	c.*35G>A	
		c.*123C>G	c.*43C>T	c.*86C>A	c.*86C>T		c.*46C>T	c.*205G>T	c.*34C>T	c.*37C>T	
		c.*138C>A	c.*43C>T	c.*106A>G	c.*93C>T		c.*54T>G		c.*34C>T	c.*37C>T	
		c.*163C>A	c.*69G>T	c.*108A>G	c.*109dupA		c.*62G>A		c.*35C>T	c.*38G>A	
			**c.*83G>T**	c.*111A>G	c.*109delA		c.*76G>A		c.*36C>T	c.*43C>T	
			**c.*83G>A**	c.*136T>G	c.*109delA		c.*76G>A		c.*37T>C	c.*56G>A	
			c.*90T>G	c.*151A>C	c.*109delA		c.*78G>A		c.*39G>T	c.*62C>T	
			c.*110C>T	c.*236delA	c.*109delA		c.*79G>A		c.*41C>G	c.*91G>T	
			c.*114C>G		c.*116C>T		c.*79G>A		c.*42C>T	c.*113T>G	
			c.*118A>T		c.*116C>T		c.*84C>T		c.*50G>A	c.*118G>T	
			c.*124C>A		c.*117G>A		c.*84C>T		c.*57C>A	c.*127G>A	
			c.*124C>A		c.*117G>A		c.*84C>T		c.*58C>T	c.*132delA	
			c.*131A>C		c.*120G>A		c.*89T>G		c.*60C>A	c.*159C>A	
			c.*209dupA				c.*91C>T		c.*61C>G	c.*184A>C	
			c.*267T>C				c.*95T>C		c.*61C>A		
							c.*109T>G		c.*62C>T		
							c.*128A>G		c.*70C>T		
							c.*136T>C		c.*78C>T		
							c.*139A>G		c.*126C>T		
							c.*233G>T		c.*135C>A		
									c.*141C>A		
									c.*190dupT		

*: stop codon
