# Supplementary material for: The Somatic Mutation Landscape of UDP-Glycosyltransferase (UGT) Genes in Human Cancers
Source: Cancers (Basel). 2022 Nov 21;14(22):5708. doi: 10.3390/cancers14225708 (PMC9688768; doi:10.3390/cancers14225708)
Supplement: Supplementary file 1 [file cancers-14-05708-s001.zip › Table S8.pdf]

Table S8. Recurrent somatic mutations at *UGT* genes in TCGA tumors

UGT genes	Twice	Three times	Four times	Five times	Six times	Eight times
1A8	c.78G>A (Gly26Gly); c.391C>T (Arg131Ter); c.392G>A (Arg131Gln); c.504C>T (Phe168Phe)	c.761G>A (Arg254Gln)				
1A10	c.78G>A (Gly26Gly); c.174C>T (Val58Val); 364delT (Ser122ArgfsTer12); c.414G>A (Lys138Lys); c.445C>T (Pro149Ser); c.474T>C (Ala158Ala); c.807G>A (Met269Ile)	c.422C>A (Ser141Tyr)<br>c.563C>T (Ser188Phe)		c.365C>T (Ser122Leu)		
1A9	c.32C>T (Pro11Leu); c.174C>T (Val58Val); 364dupT (Ser122PhefsTer8); c.442G>A (Asp148Asn); c.535G>A (Glu179Lys); c.764C>T (Thr255Met); c.765G>A (Thr255Thr); 677dupA (Asn226LysfsTer8)	364delT (Ser122GlnfsTer12)	c.761G>A (Arg254Gln)			
1A7	c.446C>T (Pro149Leu); c.563C>T (Ser188Phe); c.671dupT (Lys225GlnfsTer9); c.761G>A (Arg254Gln)	364delT (Ser122GlnfsTer12)<br>c.719C>T (Thr240Met)				
1A6	c.205G>T (Glu69Ter); c.523C>T (Pro175Ser); c.531C>T (Ser177Ser); c.696C>T (Leu232Leu)					
1A5	c.516C>T (Phe172Phe)		c.262G>A (Glu88Lys)			
1A4	517dupT (Trp173LeufsTer7); c.652C>G (Leu218Val)	c.262G>A (Glu88Lys)				c.517delT (Trp173GlyfsTer8)
1A3	c.216G>T (Glu72Asp); c.225C>T (Phe75Phe); c.471C>T (Cys157Cys);	c. 271C>T (Arg91Cys)	c.248C>T (Ser83Leu)			
1A1	c.25C>T (Arg9Cys); c.194C>T (Ser65Leu); c.303 (Glu101Asp); c.477C>T (Ile159Ile); c.513C>T (Phe171Phe); c.704C>T (Ser235Leu); c.749C>G (Ser263Cys)					
1A Exons 2-5	c.997C>T (Arg333Trp); c.1147G>A (Val383Ile); c.1356G>A (Pro452Pro); c.1431C>T (Pro477Pro); c.1441G>A (Asp481Asn); c.1474 G>A (Val492Met)	c.1019C>T (Ser340Leu);<br>c.1029G>A (Ala343/Ala)			1566delA (Arg524GlufsTer22)	
2A1	c.290delA (Asn97IlefsTer18); c.509C>T (Ser170Phe); c.538G>A (Glu180Lys); c.548G>A (Cys183Tyr)					
2A2	c.223G>A (Asp75Asn); c.586C>A (Pro196Thr); c.662delA (Asn221IlefsTer27)			c.362G>A (Gly121Glu)		
2A Exons 2-6	c.865G>A (Glu289Lys); c.975C>A (Ala325Ala); c.1341C>A (His447Gln); c.1370G>A (Arg457Gln)	c.1538C>T (Ser513Phe)<br>c.1576delA (Arg526GlufsTer57)		c.1369C>T (Arg457Ter)		
2A3	c.48C>T (Leu15Leu); c.144G>A (Val48Val); c.157G>A (Val53Ile); c.262G>A (Glu88Lys); c.919C>T (Leu307Leu); c.1016G>A (Gly339Glu); c.1538C>T (Ser513Phe); c.1502C>T (Thr501Met)	c.418G>A (Glu140Lys);<br>c.770G>A (Arg257Gln);<br>c.794C>A (Pro265His);<br>c.1369C>T (Arg457Ter)		c.342delT (Phe114LeufsTer4)		
2B4	c.17C>T (Thr6Ile); c.94G>T (Glu32Ter); c.111G>A (Met37Ile); c.130G>A (Asp44Asn); c.188C>A (Ser63Tyr); c.329C>T (Ser110Leu); c.340G>T (Glu114Ter); c.541G>A (Glu181Lys); c.652C>T (Leu218Phe); c.705C>T (Phe235Phe); c.810C>G (Leu270Leu); c.933G>A (Ser311Ser); c.977C>T (Ser326Leu); c.1329G>A (Met443Ile); c.1386C>A (Phe462Leu); c.1393G>T (Glu465Ter); c.1401C>A (Val467Val); c.1420A>G (Lys474Glu); c.1444G>A (Asp482Asn); c.1565G>A (Gly522Glu)	c.133G>A (Glu45Lys);<br>c.428C>A (Ser143Ter);<br>c.776G>A (Arg259Gln);<br>c.787G>A (Asp263Asn);<br>c.828C>T (Phe276Phe);	c.775C>T (Arg259Ter)<br>c.1406G>A (Arg469His)<br>c.1575G>A (Gly525Gly)		c.534C>T (Tyr178Tyr)	
2B7	c.463C>T (Pro155Ser); c.472G>A (Glu158Lys); c.1084C>A (Leu362Ile); c.1096C>T (Pro366Ser); c.1242A>G (Arg414Arg); c.1303G>A (Asp435Asn)	c.673G>A (Glu225Lys)				
2B10	c.68G>A (Gly23Glu); c.190G>C (Asp64His); c.258G>C (Asp120His); c.392C>T (Ser131Leu); c.796C>T (Pro266Ser); c.868G>A (Glu290Lys); c.1078G>A (Asp360Asn); c1188delT (Phe396LeufsTer10); c.1300G>T (Asp434Tyr); c.1337G>A (Arg446Lys); c.1402C>T (Arg468Cys); c.1426C>T Arg476Ter); c.1485C>T (Phe495Phe); c.1574G>A (Gly525Glu); c.1579A>G (Arg527Gly)					
2B11	c.442G>A (Val148Ile); c.485C>T (Ala162Val); c.1164C>T (Ile388Ile); c.1329G>A (Met443Ile); c.1440C>A (Ala480Ala); c.1469C>A (Ser490Tyr)	c.345C>T (Ile115Ile)	c.567C>T (Phe189Phe)<br>c.1551G>A (Trp517Ter)	c.463C>T (Pro155Ser)		
2B15	c.117G>A (Met39Ile); c.203A>C (Lys68Thr); c.520C>T (Arg174Ter); c.521G>A (Arg174Gln); c.685G>A (Asp229Asn); c.753G>A (Met251Ile); c.779G>A (Arg260Gln); c.935C>T (Ser312Leu); c.1029G>T (Lys343Asn); c.1143C>A (Ile381Ile); c.1204G>A (Asp402Asn); c.1417G>A (Gly473Arg)	c.1432C>T (Arg478Ter)	c.645G>A (Met215Ile)			
2B17	c.270delT (Phe90LeufsTer2); c.355G>A (Glu119Lys); c.508C>G (Leu170Val); c.560G>A (Gly187Glu); c.705G>A Gln235Gln); c.1101C>A (Phe360Phe); c.1357C>G (Gln453Glu); c.1378C>T (Arg460Ter)	c.273G>A (Met91Ile)<br>c.641delA (Asn214IlefsTer2				
2B28	c.57C>T (Ser19Ser); c.270C>T (Ile90Ile); c.541G>A (Glu181Lys); c.805C>T (Pro269Ser); c.866C>A (Pro289His); c.1172T>C (Val391Ala); c.1181C>T (Pro394Leu); c.1262C>T (Ser421Leu); c.1464C>A (Tyr488Ter); c.1572G>T (Lys524Asn)	c.295G>A (Asp99Asn)<br>c.329C>T (Ser110Leu)	c.352G>A (Glu118Lys)			
3A1	c.75C>T (Ile25Ile); c.242C>T (Ser81Leu); c.253C>T (His85Tyr);	c.211G>A (Glu71Lys)		c.1038G>A (Vale346Val)		

	c.447C>T (Phe149Phe); c.456C>T (Phe152Phe); c.931G>A (Glu311Lys); c.976G>A (Gly326Arg); c.1187G>A (Gly396Glu); c.1378G>A (Asp460Asn); c.1396G>T (Gly466Trp); c. 1503G>T (Trp501Cys)	c.1252G>A (Asp418Asn)		c.1139G>A (Arg380His)		
3A2	c.145C>T (His49Tyr); c.418G>A (Asp140Asn); c.452C>T (Pro151Leu); c.510C>T (Phe170Phe); c.520G>A (Glu174Lys); c.964C>T (His322Tyr); c.987G>A (Trp329Ter); c.1082C>A (Pro361Gln); c. 1252G>A (Glu418Lys); c.1272G>A (Met424Ile); c.1402G>A (Ala468Thr); c.1407G>A (Thr469Thr); c.1459G>A (Asp487Asn); c.1544G>A (Arg515His);	c.314G>A (Gly105Glu) c.1057C>T (Pro353Ser) c.1403C>T (Ala468Val)	c.1404G>A (Ala468Ala) c.1406C>T (Thr469Met)		c.1038G>A (Val346Val);	
UGT8	c.560G>A (Arg187His); c.601C>A (Leu201Ile); c.1328G>A (Arg443Gln);	c.987G>T (Lys329Asn)	c.304G>A (Glu102Lys)			
